# The length of negative proximal resection margin does not significantly influence on the prognosis of Siewert type II adenocarcinoma of esophagogastric junction with or without neoadjuvant chemotherapy

**DOI:** 10.3389/fonc.2026.1779576

**Published:** 2026-02-26

**Authors:** Zhendan Yao, Maoxing Liu, Fei Tan, Ming Cui, Jiadi Xing, Chenghai Zhang, Hong Yang, Lei Chen, Kai Xu, Xiangqian Su

**Affiliations:** 1 Key Laboratory of Carcinogenesis and Translational Research (Ministry of Education), Department IV of Gastrointestinal Cancer Center, Peking University Cancer Hospital & Institute, Beijing, China; 2 State Key Laboratory of Holistic Integrative Management of Gastrointestinal Cancers, Department of Gastrointestinal Surgery IV, Peking University Cancer Hospital & Institute, Beijing, China

**Keywords:** adenocarcinoma of esophagogastric junction, frozen section analysis, neoadjuvant chemotherapy, proximal resection margin, survival

## Abstract

**Background:**

Optimal length of proximal resection margin (PRM) for locally advanced Siewert type II adenocarcinoma of esophagogastric junction (AEG) remained undetermined. Especially, the relationship between PRM length after neoadjuvant chemotherapy (NAC) and survival were seldom reported.

**Methods:**

A total of 108 consecutive locally advanced Siewert type II AEG patients were enrolled. The clinicopathological characteristics, PRM length and survival outcomes were collected. Cox proportional hazard model was used to compare the hazard rates of survival and recurrence between patients with length above and below the cut-off value. Univariable and multivariable analyses were performed to analysis association between PRM length and prognosis.

**Results:**

The mean PRM length was 13mm (range: 1–45 mm). PRM status was independent factor for recurrence-free survival (RFS) (HR 3.177, 95%CI 1.098-9.193, *p* = 0.033). 4 patients (3.7%) had positive PRM on histological pathology, and they suffered shorter RFS than patients with negative PRM (16.0 ± 4.3 months vs 60.1 ± 3.9 months, *p* = 0.002). In 104 patients with negative PRM, NAC was administered to 53 patients (51.0%). The length of PRM was not associated with survival outcomes in NAC group and surgery alone (SA) group, respectively. The hazard rates of survival and recurrence did not differ between the patients with length of PRM above and below the cut-off value (*p*>0.05). No statistically significant differences in survival outcomes were observed between patients with different PRM lengths in either the NAC group or the SA group. Similarly, no statistically significant differences in survival outcomes were found across different PRM lengths, no matter which treatment strategy was chosen.

**Conclusions:**

A positive status of PRM appears to be associated with adverse survival outcomes of patients with locally advanced Siewert type II AEG after surgery. However, for patients with negative status, the length of the PRM does not influence survival, regardless of whether they undergo surgical resection alone or NAC followed by surgery.

## 1 Introduction

In recent years, the incidence of adenocarcinoma of esophagogastric junction (AEG) has been increasing progressively (1). Radical resection serves as the cornerstone for long-term survival in patients with AEG. It is well-established that achieving a negative proximal resection margin (PRM) and performing thorough lymphadenectomy are critical components of radical resection. It has long been recognized that a positive PRM significantly compromises survival for patients with AEG (2, 3). Therefore, to ensure a negative PRM, studies have been conducted to determine the maximum distance of tumor infiltration toward the oral side, which serves as the standard for determining the length of distal esophageal resection during surgery. However, due to inevitable heterogeneity in patients enrolled across different centers, conclusions from various studies have been inconsistent. For instance, Ito H et al. (4) recommended a PRM length of 6 cm for T3/T4 patients to achieve pathological negativity, while Mariette et al. (2) suggested a length of 8 cm, and Papchristou (5) advocated for a length of up to 12 cm. Furthermore, some authors observed local recurrence in advanced patients even with negative PRM, hypothesizing that this might be due to insufficient length of PRM (6). Consequently, they recommended increasing the length even when the PRM was negative, in order to reduce the risk of recurrence.

According to Siewert classification, AEG is divided into three subtypes (7). Siewert type I refers to tumors whose center located 1-5cm above the esophagogastric junction (EGJ), primarily operated on via trans-thoracic approach; Siewert type III involves tumors with center 2-5cm below the EGJ, mainly managed through trans-abdominal approach. In contrast, Siewert type II AEG, defined as tumors with center located between 1 cm above and 2 cm below the EGJ, had been subject to considerable debate regarding the optimal surgical approach (8–10). Historically, surgeons opted for transthoracic approach for Siewert type II AEG to achieve adequate length of PRM mentioned above. However, studies by Hulscher et al. (11) and the Japan Clinical Oncology Group (JCOG) 9502 trial (12) found no significant difference in long-term survival between transthoracic and abdominal-transhiatal approaches, besides, the transthoracic approach associated with poorer short-term recovery. A meta-analysis of 1,155 patients included 8 studies further confirmed both approaches yield comparable long-term survival, but the abdominal-transhiatal approach significantly reduced 30-day mortality, pulmonary complications, and shortened hospital stays (13). Meanwhile, Kurokawa Y et al. (14) discovered that Siewert type II AEG patients with esophageal invasion exceeding 3cm had a higher rate of mediastinal lymph node metastasis, and transthoracic lymphadenectomy could offer survival benefits in such cases. Therefore, for patients with Siewert type II AEG invading the esophagus less than 3cm, the abdominal-transhiatal approach is recommended as the standard surgical procedure. Nevertheless, it is undeniable that the PRM length is closely related to the surgical approach, and the length of PRM is restricted by the limited subdiaphragmatic space in the abdominal-transhiatal approach. Kinoshita T et al. (15) reported a median length of PRM in this procedure was 14.5mm (range 10-23mm). While Sugita S et al. (16) observed a median of only 9mm (range 3-26mm) in laparoscopic-assisted abdominal-transhiatal procedure. Consequently, whether the length of PRM in abdominal-transhiatal approach for Siewert type II AEG affects survival and prognosis remains a key clinical concern.

In China, locally advanced AEG accounts for a considerable proportion. According to guidelines recommendations, perioperative chemotherapy combined with surgical resection is the standard treatment strategy for locally advanced AEG (17, 18). However, many previous studies focused on the safe length of PRM for AEG have excluded patients receiving neoadjuvant chemotherapy (NAC) (19–22), and thus the optimal PRM length for Siewert type II AEG patients after NAC remains undetermined. Accordingly, we conducted this study utilizing a laparoscopic-assisted abdominal-transhiatal approach to treat patients with locally advanced Siewert type II AEG. The primary objective was to investigate the association between PRM length and survival outcomes, with particular emphasis on determining the optimal PRM length following NAC.

## 2 Materials and methods

### 2.1 Patients

**Figure 1:**
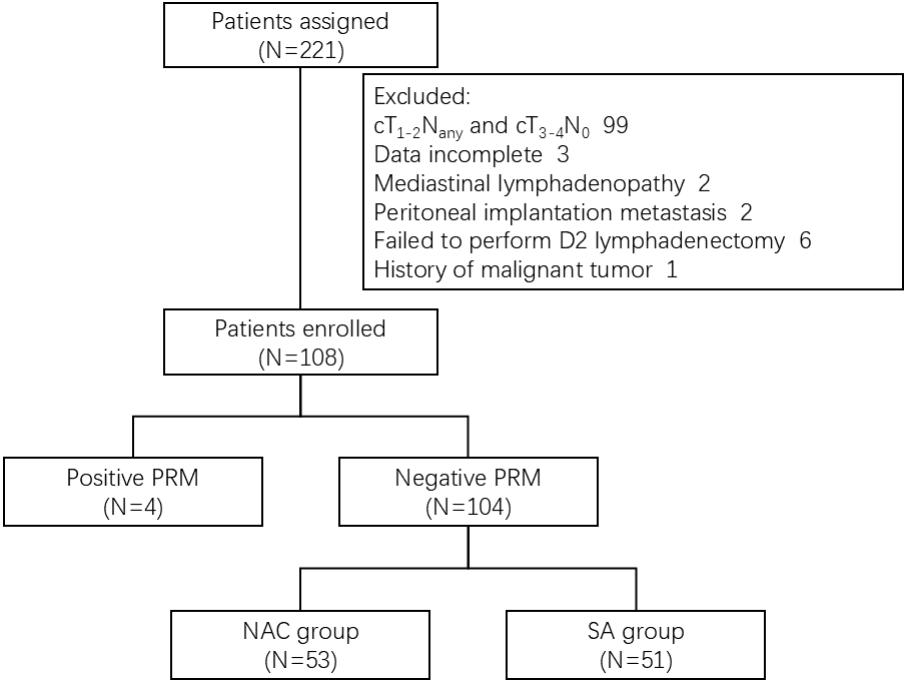
Flow diagram of treatment patterns. PRM, proximal resection margin; NAC, neoadjuvant chemotherapy; SA, surgery alone.

From June 2015 to December 2020, a total of 221 consecutive patients with Siewert type II AEG underwent surgical treatment at Ward IV of the Gastrointestinal Oncology Center, Peking University Cancer Hospital. The inclusion criteria for this study were: Eastern Cooperative Oncology Group (ECOG) performance score ≤1; gastroscopy indicating Siewert type II tumors with esophageal invasion ≤3cm and histopathological confirmation of adenocarcinoma; preoperative imaging excluding distant metastases in liver, lung, and Virchow lymph nodes; preoperative clinical staging cT_3-4_N_1-3_M_0_. Exclusion criteria included: inability to accurately provide PRM length; preoperative imaging showing mediastinal lymphadenopathy; intraoperative laparoscopic exploration indicating peritoneal implantation metastasis; positive intraoperative lavage cytology; failure to complete D2 lymphadenectomy; and history of other malignancies. Ultimately, 108 patients were enrolled in this study (**Figure 1**). All of the patients enrolled provided written informed consent.

### 2.2 Treatment strategy

The treatment protocol for locally advanced AEG was primarily established through multidisciplinary-team (MDT) discussions. Patients were fully informed of the potential benefits and risks for NAC, after which they made a decision regarding acceptance of NAC or proceeding directly to surgical resection. The most commonly employed chemotherapy regimens included capecitabine plus oxaliplatin (CapeOX) and S-1 plus oxaliplatin (SOX). For patients undergoing NAC, tumor response was evaluated every two cycles using plain chest computed tomography (CT) and contrast-enhanced abdominal CT scans. If radical surgical resection became feasible following 2–4 cycles, surgical resection was promptly performed.

At the initiation of surgery, laparoscopic exploration was performed to rule out peritoneal metastasis, and lavage cytology was conducted to exclude cytological positivity. In cases where no peritoneal metastasis was detected and lavage cytology was negative, radical resection was undertaken. The standard surgical strategy consisted of laparoscopic-assisted total gastrectomy with transhiatal resection of the distal esophagus and Roux-en-Y esophagojejunostomy. After complete mobilization of the stomach laparoscopically, a 12-15cm upper abdominal incision was made. The surgeon identified the upper border of the tumor by palpation and then transected the esophagus at a safe distance. In cases of significant tumor regression or uncertain tumor localization, intraoperative gastroscopy was utilized to accurately determine the tumor extent. Immediately after specimen removal, the gastric wall was opened to measure the distance between the PRM and upper border of the tumor. If the length exceeded 2cm and visual inspection of the mucosal margin showed no abnormalities, frozen section analysis (FSA) was not mandatory; otherwise, FSA was carried out. Furthermore, if the FSA result was negative, the subsequent surgical procedure would proceed as planned. If positive, an additional 1-2cm of distal esophagus was resected and send for repeat FSA until negative results were confirmed. Adjuvant chemotherapy was initiated within 45 days postoperatively, based on pathological findings and the patient’s recovery status. Adjuvant chemotherapy regimens included CapeOX, SOX, or monotherapy with S-1.

### 2.3 Follow-up

In this study, patients were followed up every 3 months within the first 2 years postoperatively, every 6 months from the 3^rd^ to 5^th^ year, and every 12 months after 5 years.

The deadline of the follow-up was December 31, 2024. Recurrence-free survival (RFS) was defined as the period from surgery to the first recurrence and/or death; overall survival (OS) was defined as the duration from surgery to either death or the last follow-up.

The primary endpoint of this study was 5-year RFS rate, while the secondary endpoint was 5-year OS rate.

### 2.4 Data collection

In this study, data were retrospectively collected from the electronic medical record system, including patient demographics, imaging and endoscopic examinations, preoperative tumor markers [carcinoembryonic antigen (CEA), carbohydrate antigen 19-9 (CA19-9)], pathological characteristics [tumor location, size, gross type, differentiation, pathological Tumor-Node-Metastasis (pTNM) stage, PRM length], as well as follow-up information (date of first recurrence, date of death).

The PRM length was defined as the distance from the uppermost edge of the tumor to the esophageal resection margin, measured in millimeters microscopically after formalin fixation of the specimen (23). For patients with intraoperative FSA showing PRM involved, additional esophageal resection was performed, and the length of the resected segment was added to the PRM length (19, 20, 22). In cases where esophagojejunostomy was performed using a circular stapler, the thickness of the doughnut was not included in the PRM length (24). All length measurements mentioned above were independently performed by two pathologists. If the length error was within 10%, the average value was recorded. However, when the length error exceeded 10%, a third pathologist verified and determined the final measurement.

### 2.5 Statistical analysis

IBM SPSS Statistics (Version 25.0; IBM Corp., New York, USA) was applied for data statistical analysis. Data were presented as the x ± s for continuous variables and as numbers and percentages for categorical variables. Differences between groups were calculated using the independent t-test, X2 test or Fisher’s exact test, as appropriate. Especially, PRM length was analyzed both as a continuous variable (in millimeters) in Cox regression models and as a categorical variable using predefined cutoff values for subgroup comparisons. Cumulative RFS and OS rates were compared using the Kaplan-Meier method and Log-rank test. Cox proportional regression model was used for multivariable prognostic analysis. Multivariate logistic regression analysis was used for all variables with *p* < 0.10 on univariate analysis. *P* < 0.05 was considered statistically significant.

## 3 Results

### 3.1 Clinical and pathological characteristics of all patients

From June 2015 to December 2020, a total of 108 patients with locally advanced Siewert type II AEG were included in this retrospective study. Among them, 87 patients (80.6%) were male, with a median age of 60 years (range: 22-80). NAC was administered to 53 patients (49.1%). The mean PRM length was 13mm (range: 1–45 mm). FSA was carried out in 93 patients (86.1%), of which 5 patients showed positive on FSA examination. All these 5 patients underwent intraoperative additional esophageal extended resection, and the histological pathology results confirmed negative. Additionally, other 4 patients (3.7%) had positive PRM on histology. The median follow-up period for all patients was 52 months (range: 5-105), calculated using the reverse Kaplan-Meier method. The follow-up completeness rate was 98.1% (106/108). During the follow-up period, 57 patients (52.8%) experienced recurrence and 50 patients (46.3%) died.

### 3.2 Survival status and influencing factors of all patients

#### 3.2.1 RFS for all patients

The median RFS for the entire cohort was 58.5 ± 3.9 months, with cumulative RFS rates of 73%, 57%, and 46% at 1-,3-, and 5-year, respectively. Univariable analysis indicated that lymph-vascular invasion (HR 2.01, 95%CI 1.163-3.473, *p* = 0.012), PRM status (HR 4.346, 95%CI 1.529-12.348, *p* = 0.006), pN stage (HR 2.156, 95%CI 1.270-3.660, *p* = 0.004), and pTNM stage (HR 2.181, 95%CI 1.269-3.750, *p* = 0.005) were potentially associated with RFS in the overall patient. These variables, along with other factors with *p* < 0.1, were included in multivariable analysis, which revealed that PRM status (HR 3.177, 95%CI 1.098-9.193, *p* = 0.033) and pTNM stage (HR 2.032, 95%CI 1.68-3.534, *p* = 0.012) were independent factors for RFS (**Table 1**).

**Table 1:** Univariable and multivariable analysis of risk factors for RFS and OS of overall patients.

Clinicopathologic features	Univariable analysis for RFS (HR, 95%*CI*, *p* value)	Multivariable analysis for RFS (HR, 95%*CI*, *p* value)	Univariable analysis for OS (HR, 95%*CI*, *p* value)	Multivariable analysis for OS (HR, 95%*CI*, *p* value)
Gender (Male/Female)	0.633 (0.300-1.338, 0.231)		0.519 (0.221-1.218, 0.132)	
Age	1.008 (0.982-1.035, 0.534)		1.011 (0.982-1.040, 0.461)	
NAC (No/Yes)	0.633 (0.374-1.071, 0.088)	1.265 (0.641-2.493, 0.498)	0.698 (0.398-1.225, 0.210)	
CEA (Normal/abnormal)	1.258 (0.711-2.224, 0.430)		1.206 (0.657-2.216, 0.545)	
CA19_9 (Normal/abnormal)	1.462 (0.754-2.831, 0.261)		1.831 (0.909-3.688, 0.090)	2.115 (1.038-4.312, 0.039)
Borrmann type (Type 1+2/ Type 3+4)	2.116 (0.765-5.853, 0.149)		1.704 (0.613-4.737, 0.307)	
Length of PRM	0.830 (0.585-1.177, 0.296)		0.948 (0.663-1.356, 0.769)	
Mass size (<5 cm/≥5 cm)	1.611 (0.950-2.731, 0.077)	1.340 (0.750-2.396, 0.323)	1.505 (0.858-2.641, 0.154)	
Differentiation (Well/Poor)	0.971 (0.576-1.637, 0.912)		0.797 (0.457-1.391, 0.425)	
Lauren classification (Intestinal/ Others)	0.876 (0.598-1.284, 0.497)		0.725 (0.409-1.285, 0.271)	
VLI (Negative/Positive)	2.010 (1.163-3.473, 0.012)	1.399 (0.714-2.743, 0.328)	1.560 (0.881-2.765, 0.127)	
Perineural invasion (Negative/Positive)	1.452 (0.837-2.519, 0.185)		1.300 (0.723-2.335, 0.381)	
Status of PRM (Negative/Positive)	4.346 (1.529-12.348, 0.006)	3.177 (1.098-9.193, 0.033)	2.516 (0.902-7.018, 0.078)	2.046 (0.705-5.938, 0.188)
pT (pT0-2/ pT3-4)	2.770 (0.865-8.868, 0.086)	1.666 (0.477-5.820, 0.424)	2.119 (0.659-6.815, 0.208)	
pN (pN0-1/pN2-3)	2.156 (1.270-3.660, 0.004)	1.213 (0.344-4.290, 0.763)	1.983 (1.124-3.499, 0.018)	2.152 (1.205-3.843, 0.010)
pTNM (pTNM 0-2/pTNM 3)	2.181 (1.269-3.750, 0.005)	2.032 (1.168-3.534, 0.012)	2.003 (1.122-3.577, 0.019)	1.811 (0.412-7.956, 0.432)

RFS, Recurrence-free survival; OS, Overall survival; NAC, Neoadjuvant chemotherapy; CEA, carcinoembryonic antigen; CA19_9, carbohydrate antigen 19-9; PRM, proximal resection margin; VLI, Vascular-lymphatic invasion; pTNM, pathologic tumor-node-metastasis staging; HR, hazard ratio; CI, confidence interval.

*PRM length was analyzed as a continuous variable.

#### 3.2.2 OS for all patients

The median OS for all patients was 69.3 ± 3.7 months, with 1-,3-, and 5-year cumulative OS rates of 91%,70%, and 53%, respectively. Univariable analysis indicated that pN (HR 1.983, 95%CI 1.124-3.499, *p* = 0.018) and pTNM staging (HR 2.003, 95%CI 1.122-3.577, *p* = 0.019) might be associated with OS in the overall patients. When these variables were included in multivariable analysis along with other *p* < 0.1 factors, preoperative CA19–9 levels (HR 2.115, 95%CI 1.038-4.312, *p* = 0.039) and pN stage (HR 2.152, 95%CI 1.205-3.843, *p* = 0.01) identified as independent factors for OS of all patients (**Table 1**).

### 3.3 Comparison of clinicopathological characteristics and survival outcomes by different PRM statuses

**Figure 2:**
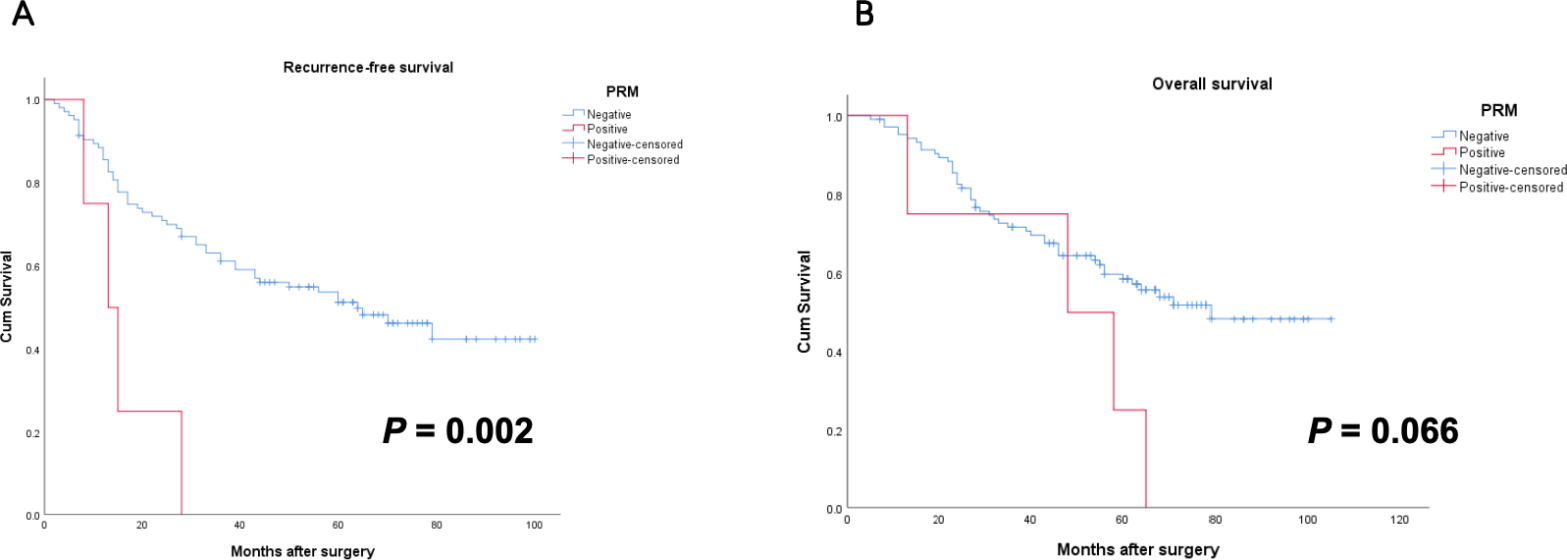
Survival curves for Siewert type II AEG patients with positive and negative PRM. **(A)** Patients with positive PRM had worse RFS than patients with negative PRM (*p*=0.002); **(B)** There was no statistically significant difference between OS of patients with positive PRM and negative PRM (*p*=0.066). AEG, adenocarcinoma of esophagogastric junction; PRM, proximal resection margin; RFS, recurrence-free survival; OS, overall survival.

In this cohort, 4 patients had positive status of PRM on histological pathology. Compared to those with negative PRM, these patients exhibited significantly more advanced pN and pTNM stages, with statistically significant differences. However, no statistically significant difference was observed in the length of PRM between the two groups (**Table 2**). In terms of survival, the patients with positive PRM had shorter RFS (16.0 ± 4.3 months) and OS (46.0 ± 11.5 months) than the negative PRM (RFS: 60.1 ± 3.9 months; OS: 70.5 ± 3.8 months). The difference in RFS was statistically significant (*p* = 0.002) (**Figure 2**).

**Table 2:** Comparison of clinicopathological characteristics and survival outcomes by patients with negative and positive status of PRM.

Variable	PRM negative (n=104) (%)	PRM positive (n=4) (%)	*P* value	Variable	PRM negative (n=104) (%)	PRM positive (n=4) (%)	*P* value
Gender			0.775	Lauren classification			0.166
Male	84 (80.8)	3 (75.0)		Intestinal	61 (59.8)	1 (25.0)	
Female	20 (19.2)	1 (25.0)		Others	41 (40.2)	3 (75.0)	
Age	60.8±10.4	60.3±8.3	0.907	VLI			0.054
NAC			0.136	Negative	51 (49.0)	0 (0.0)	
No	51 (49.0)	4 (100.0)		Positive	53 (51.0)	4 (100.0)	
Yes	53 (51.0)	0 (0.0)		Perineural invasion			0.111
CEA			0.914	Negative	41 (39.4)	0 (0.0)	
Normal	74 (72.5)	3 (75.0)		Positive	63 (60.0)	4 (100.0)	
Abnormal	28 (27.5)	1 (25.0)		pT			0.471
CA19_9			0.619	pT0-2	12 (11.5)	0 (0.0)	
Normal	86 (84.3)	3 (75.0)		pT3-4	92 (88.5)	4 (100.0)	
Abnormal	16 (15.7)	1 (25.0)		pN			0.028
Borrmann type			0.493	pN0-1	58 (55.8)	0 (0.0)	
Type 1+2	11 (10.6)	0 (0.0)		pN2-3	46 (44.2)	4 (100.0)	
Type 3+4	93 (89.4)	4 (100.0)		pTNM			0.050
Length of PRM	1.3±0.8	1.2±0.8	0.717	pTNM0-2	52 (50.0)	0 (0.0)	
Mass size			0.080	pTNM3	52 (50.0)	4 (100.0)	
<5 cm	70 (67.3)	1 (25.0)		5 Y Survival rate (%)	63 (60.0)	1 (25.0)	0.155
≥5 cm	34 (32.7)	3 (75.0)		5 Y Recurrence-free rate (%)	55 (52.9)	0 (0.0)	0.038
Differentiation			0.384	OS (m)	70.5±3.8	46.0±11.5	0.066
Well	49 (47.1)	1 (25.0)		RFS (m)	60.1±3.9	16.0±4.3	0.002
Poor	55 (52.9)	3 (75.0)					

PRM, proximal resection margin; NAC, Neoadjuvant chemotherapy; CEA, carcinoembryonic antigen; CA19_9, carbohydrate antigen 19-9; VLI, Vascular-lymphatic invasion; pTNM, pathologic tumor-node-metastasis staging; OS, Overall survival; RFS, Recurrence-free survival.

### 3.4 Comparison of clinicopathological characteristics and survival outcomes by different treatment strategy in patients with negative PRM

Subsequently, 4 patients with positive PRM status on histological pathology were excluded. Among the rest 104 patients, 53 (51.0%) underwent NAC followed by surgical resection (NAC group). Compared to those who underwent surgical resection alone (SA group), NAC group demonstrated significantly smaller tumor size, lower rates of lymph-vascular invasion and perineural invasion, and earlier pTNM staging (all *p* < 0.05). There was no statistically significant difference in PRM length between these two groups (NAC group: 13.6 ± 7.1 mm vs. SA group: 12.4 ± 8.8 mm, *p* = 0.439). Although NAC group showed longer RFS and OS than SA group (RFS: 64.6 ± 5.3 vs. 54.7 ± 5.6, *p* = 0.185; OS: 71.1 ± 4.9 vs. 67.1 ± 5.5, *p* = 0.344), the differences were not statistically significant (**Table 3**).

**Table 3:** Comparison of clinicopathological characteristics and survival outcomes by patients with negative status of PRM in SA and NAC group.

Variable	SA (n=51) (%)	NAC (n=53) (%)	*P* value	Variable	SA (n=51) (%)	NAC (n=53) (%)	*P* value
Gender			0.688	VLI			<0.001
Male	42 (82.4)	42 (79.2)		Negative	15 (29.4)	36 (67.9)	
Female	9 (17.6)	11 (20.8)		Positive	36 (70.6)	17 (32.1)	
Age	61.5±11.4	60.3±9.4	0.576	Perineural invasion			0.004
CEA			0.841	Negative	13 (25.5)	28 (52.8)	
Normal	36 (73.5)	38 (71.7)		Positive	38 (74.5)	25 (47.2)	
Abnormal	13 (26.5)	15 (28.3)		pT			0.003
CA19_9			0.474	pT0-2	1 (2.0)	11 (20.8)	
Normal	40 (81.6)	46 (86.8)		pT3-4	50 (98.0)	42 (79.2)	
Abnormal	9 (18.4)	7 (13.2)		pN			<0.001
Borrmann type			0.306	pN0-1	16 (31.4)	42 (79.2)	
Type 1+2	7 (13.7)	4 (7.5)		pN2-3	35 (68.6)	11 (20.8)	
Type 3+4	44 (86.3)	49 (92.5)		pTNM			<0.001
Length of PRM	1.24±0.88	1.36±0.71	0.439	pTNM0-2	12 (23.5)	40 (75.5)	
Mass size			0.008	pTNM3	39 (76.5)	13 (24.5)	
<5 cm	28 (54.9)	42 (79.2)		5 Y Survival rate (%)	28 (54.9)	35 (66.0)	0.245
≥5 cm	23 (45.1)	11 (20.8)		5 Y Recurrence-free rate (%)	22 (43.1)	33 (62.3)	0.051
Differentiation			0.991	OS (m)	67.1±5.5	71.1±4.9	0.344
Well	24 (47.1)	25 (47.2)		RFS (m)	54.7±5.6	64.6±5.3	0.185
Poor	27 (52.9)	28 (52.8)					
Lauren classification			0.545				
Intestinal	29 (56.9)	32 (62.7)					
Others	22 (43.1)	19 (37.3)					

PRM, proximal resection margin; SA, Surgery alone; NAC, Neoadjuvant chemotherapy; CEA, carcinoembryonic antigen; CA19_9, carbohydrate antigen 19-9; VLI, Vascular-lymphatic invasion; pTNM, pathologic tumor-node-metastasis staging; OS, Overall survival; RFS, Recurrence-free survival.

### 3.5 The relationship between PRM length and survival outcomes in patients with negative PRM

Among 104 patients with negative PRM status, a Cox proportional hazards model was used to analyze factors for RFS and OS. Univariate analysis indicated that the length of PRM was not associated with survival outcomes in the entire cohort (RFS: HR 0.740, 95% CI 0.450–1.214, *p* = 0.233; OS: HR 0.923, 95% CI 0.566–1.505, *p* = 0.748), in NAC group (RFS: HR 0.919, 95% CI 0.423–1.997, *p* = 0.832; OS: HR 0.987, 95% CI 0.449–2.169, *p* = 0.973), or in SA group (RFS: HR 0.657, 95% CI 0.339–1.271, *p* = 0.212; OS: HR 0.911, 95% CI 0.487–1.705, *p* = 0.771) (**Tables 4A**, **B**).

**Table 4A:** Univariable and multivariable analysis of risk factors for RFS of patients with negative status of PRM in different treatment group.

Clinicopathologic features	Univariable analysis for all patients (HR, 95%*CI*, *p* value)	Multivariable analysis for all patients (HR, 95%*CI*, *p* value)	Univariable analysis for NAC patients (HR, 95%*CI*, *p* value)	Multivariable analysis for NAC patients (HR, 95%*CI*, *p* value)	Univariable analysis for SA patients (HR, 95%*CI*, *p* value)	Multivariable analysis for SA patients (HR, 95%*CI*, *p* value)
Gender (Male/Female)	0.445 (0.161-1.227), 0.118		0.375 (0.087-1.612), 0.187		0.544 (0.127-2.324), 0.411	
Age	1.007 (0.970-10.045), 0.703		1.052 (0.985-1.124), 0.131		0.977 (0.928-1.028), 0.371	
NAC (No/Yes)	0.628 (0.289-1.362), 0.239					
CEA (Normal/abnormal)	1.407 (0.586-3.381),0.445		2.300 (0.679-7.796), 0.181		0.833 (0.233-2.985), 0.779	
CA19_9 (Normal/abnormal)	1.746 (0.583, 5.229), 0.319		0.436 (0.077-2.483), 0.350		8.000 (0.914-70.016), 0.060	11.486 (1.110-118.903), 0.041
Borrmann type (Type 1+2/3+4)	1.949 (0.534-7.109), 0.312		2.654 (0.258-27.322), 0.412		1.926 (0.384-9.665), 0.426	
Length of PRM	0.740 (0.450-1.214), 0.233		0.919 (0.423-1.997), 0.832		0.657 (0.339-1.271), 0.212	
Mass size (<5 cm/≥5 cm)	1.918 (0.831-4.427), 0.127		2.574 (0.651-10.172), 0.178		1.348 (0.440-4.129), 0.601	
Differentiation (Well/Poor)	0.996 (0.461-2.151), 0.991		1.500 (0.504-4.463), 0.466		0.646 (0.211-1.979), 0.444	
Lauren classification (Intestinal/ Others)	0.808 (0.366-1.786), 0.598		0.727 (0.232-2.284), 0.585		0.847 (0.277-2.591), 0.771	
VLI (Negative/Positive)	2.557 (1.160-5.637), 0.020	1.788 (0.716-4.464), 0.213	3.244 (0.972-10.823), 0.056	1.946 (0.503-7.531), 0.335	1.796 (0.532-6.057), 0.345	
Perineural invasion (Negative/Positive)	1.358 (0.617-2.988), 0.448		1.231 (0.416-3.640), 0.707		1.179 (0.332-4.182), 0.799	
pT (pT0-2/ pT3-4)	3.571 (0.908-14.048), 0.068	2.214 (0.502-9.772), 0.294	4.950 (0.953-25.710), 0.057	3.349 (0.608-18.434), 0.165		
pN (pN0-1/pN2-3)	2.417 (1.092-5.350), 0.030	1.160 (0.190-7.088), 0.872	4.333 (1.001-18.767), 0.050	2.419 (0.480-12.195), 0.284	1.500 (0.456-4.934), 0.504	
pTNM (pTNM 0-2/pTNM 3)	2.363 (1.075-5.187),0.032	1.299 (0.221-7.634), 0.772	2.400 (0.665-8.666), 0.181		2.240 (0.600-8.356), 0.230	

RFS, Recurrence-free survival; PRM proximal resection margin; NAC, Neoadjuvant chemotherapy; CEA, carcinoembryonic antigen; CA19_9, carbohydrate antigen 19-9; VLI, Vascular-lymphatic invasion; SA, Surgery alone; pTNM, pathologic tumor-node-metastasis staging; HR, hazard ratio; CI, confidence interval.

*PRM length was analyzed as a continuous variable.

**Table 4B:** Univariable and multivariable analysis of risk factors for OS of patients with negative status of PRM in different treatment group.

Clinicopathologic features	Univariable analysis for all patients (HR, 95%*CI*, *p* value)	Multi variable analysis for all patients (HR, 95%*CI*, *p* value)	Univariable analysis for NAC patients (HR, 95%*CI*, *p* value)	Multivariable analysis for NAC patients (HR, 95%*CI*, *p* value)	Univariable analysis for SA patients (HR, 95%*CI*, *p* value)	Multivariable analysis for SA patients (HR, 95%*CI*, *p* value)
Gender (Male/Female)	0.350 (0.117-1.049), 0.061	0.296 (0.093-0.942), 0.039	0.269 (0.052-1.398), 0.118		0.455 (0.100-2.063), 0.307	
Age	1.011 (0.974-1.050), 0.566		1.047 (0.979-1.120), 0.180		0.989 (0.942-1.038), 0.651	
NAC (No/Yes)	0.683 (0.314-1.485), 0.336					
CEA (Normal/abnormal)	1.387 (0.579-3.321), 0.463		2.198 (0.652-7.413), 0.204		0.857 (0.240-3.056), 0.812	
CA19_9 (Normal/abnormal)	1.786 (0.608-5.241), 0.291		0.217 (0.024-1.947), 0.172		12.000 (1.366-105.412), 0.025	19.200 (1.717-214.689), 0.016
Borrmann type (Type 1+2/3+4)	1.949 (0.534-7.109), 0.312		2.654 (0.258-27.322), 0.412		1.926 (0.384-9.665), 0.426	
Length of PRM	0.923 (0.566-1.505), 0.748		0.987 (0.449-2.169), 0.973		0.911 (0.487-1.705), 0.771	
Mass size (<5 cm/≥5 cm)	1.687 (0.739, 3.854), 0.214		2.160 (0.563-8.284), 0.261		1.259 (0.417-3.800), 0.683	
Differentiation (Well/Poor)	0.813 (0.374-1.765), 0.600		0.971 (0.322-2.923), 0.958		0.677 (0.224-2.044), 0.489	
Lauren classification (Intestinal/ Others)	0.661 (0.296-1.477), 0.313		0.523 (0.159-1.721), 0.286		0.778 (0.256-2.363), 0.658	
VLI (Negative/Positive)	1.749 (0.800-3.825), 0.161		2.250 (0.693-7.306), 0.177		1.143 (0.342-3.819), 0.828	
Perineural invasion (Negative/Positive)	1.204 (0.544-2.666), 0.647		1.030 (0.342-3.103), 0.958		1.167 (0.330-4.122), 0.811	
pT (pT0-2/ pT3-4)	2.633 (0.669-10.353), 0.166		3.717 (0.715-19.323), 0.118			
pN (pN0-1/pN2-3)	2.470 (1.115-5.473), 0.026	2.170 (0.324-14.549), 0.425	5.949 (1.355-26.117), 0.018	4.965 (1.085-22.732), 0.039	1.361 (0.414, 4.473), 0.611	
pTNM (pTNM 0-2/pTNM 3)	2.204 (1.000-4.854), 0.050	1.121 (0.168-7.488), 0.906	3.323 (0.907-12.176), 0.070	1.353 (0.700-2.616), 0.368	1.474 (0.398-5.452), 0.561	

OS, Overall survival; PRM, proximal resection margin; NAC, Neoadjuvant chemotherapy; CEA, carcinoembryonic antigen; CA19_9, carbohydrate antigen 19-9; VLI, Vascular-lymphatic invasion; pTNM, pathologic tumor-node-metastasis staging; HR, hazard ratio; CI, confidence interval; SA, Surgery alone. *PRM length was analyzed as a continuous variable.

In this study, we stratified PRM lengths using 5 mm, 10 mm, and 20 mm as cutoff values (25) and employed the COX proportional hazards model to compare hazard ratios among different length groups, thereby investigating the impact of PRM length on recurrence and survival. The results revealed that among patients with negative PRM status, there were no statistically significant differences in the risks of recurrence and survival outcomes across the various PRM length cutoff values (**Table 5**).

**Table 5:** Overall appearance of adjusted hazard ratio.

Patients type	Cut-off value	HR (95%*CI*, *p* value)-OS	HR (95%*CI*, *p* value)-RFS
All	5mm	0.849 (0.430-1.677, 0.637)	0.754 (0.403-1.412, 0.378)
	10mm	1.110 (0.621-1.983, 0.724)	0.801 (0.544-1.600, 0.801)
	20mm	1.092 (0.565-2.111, 0.793)	0.820 (0.431-1.562, 0.547)
NAC	5mm	0.557 (0.203-1.529, 0.256)	0.660 (0.245-1.777, 0.411)
	10mm	1.461 (0.602-3.549, 0.402)	1.549 (0.674-3.561, 0.303)
	20mm	1.393 (0.538-3.605, 0.494)	1.056 (0.418-2.669, 0.908)
SA	5mm	1.213 (0.483-3.045, 0.680)	0.892 (0.395-2.017, 0.784)
	10mm	0.918 (0.416-2.025, 0.833)	0.638 (0.301-1.352, 0.241)
	20mm	0.877 (0.350-2.197, 0.779)	0.658 (0.268-1.617, 0.361)

NAC, Neoadjuvant chemotherapy; SA, Surgery alone; HR, hazard ratio; CI, confidence interval; OS, Overall survival; RFS, Recurrence-free survival.

*PRM length was analyzed as a categorical variable.

**Figure 3:**
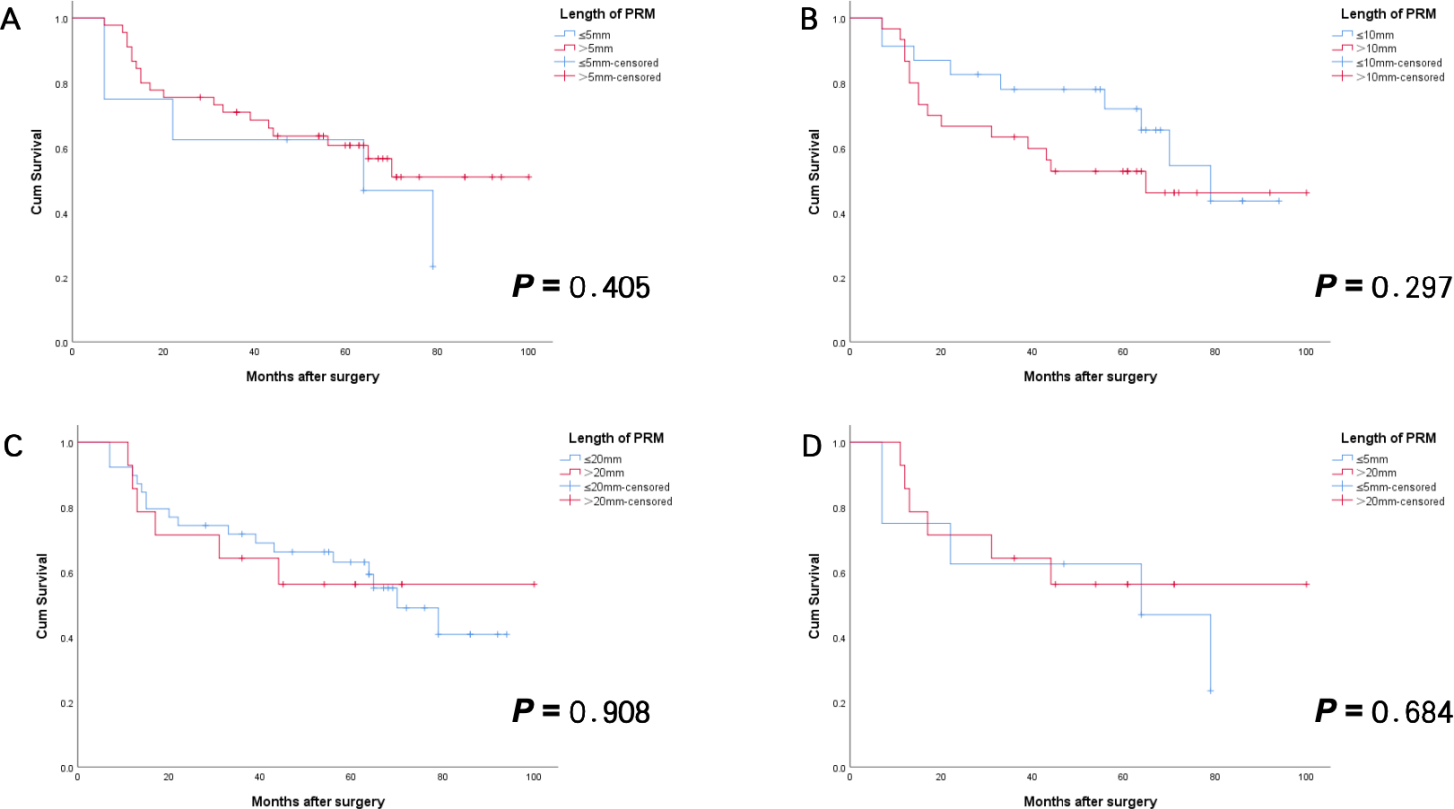
RFS curves for Siewert type II AEG patients with negative PRM in NAC group. There was no statistically significant difference between patients with PRM length above and below the different cutoff values [**(A)** ≤5mm vs > 5mm; **(B)** ≤10mm vs > 10mm; **(C)** ≤20mm vs > 20mm; **(D)** ≤5mm vs > 20mm]. (All *p*>0.05)]. AEG, adenocarcinoma of esophagogastric junction; NAC, neoadjuvant chemotherapy; PRM, proximal resection margin; RFS, recurrence-free survival.

**Figure 4:**
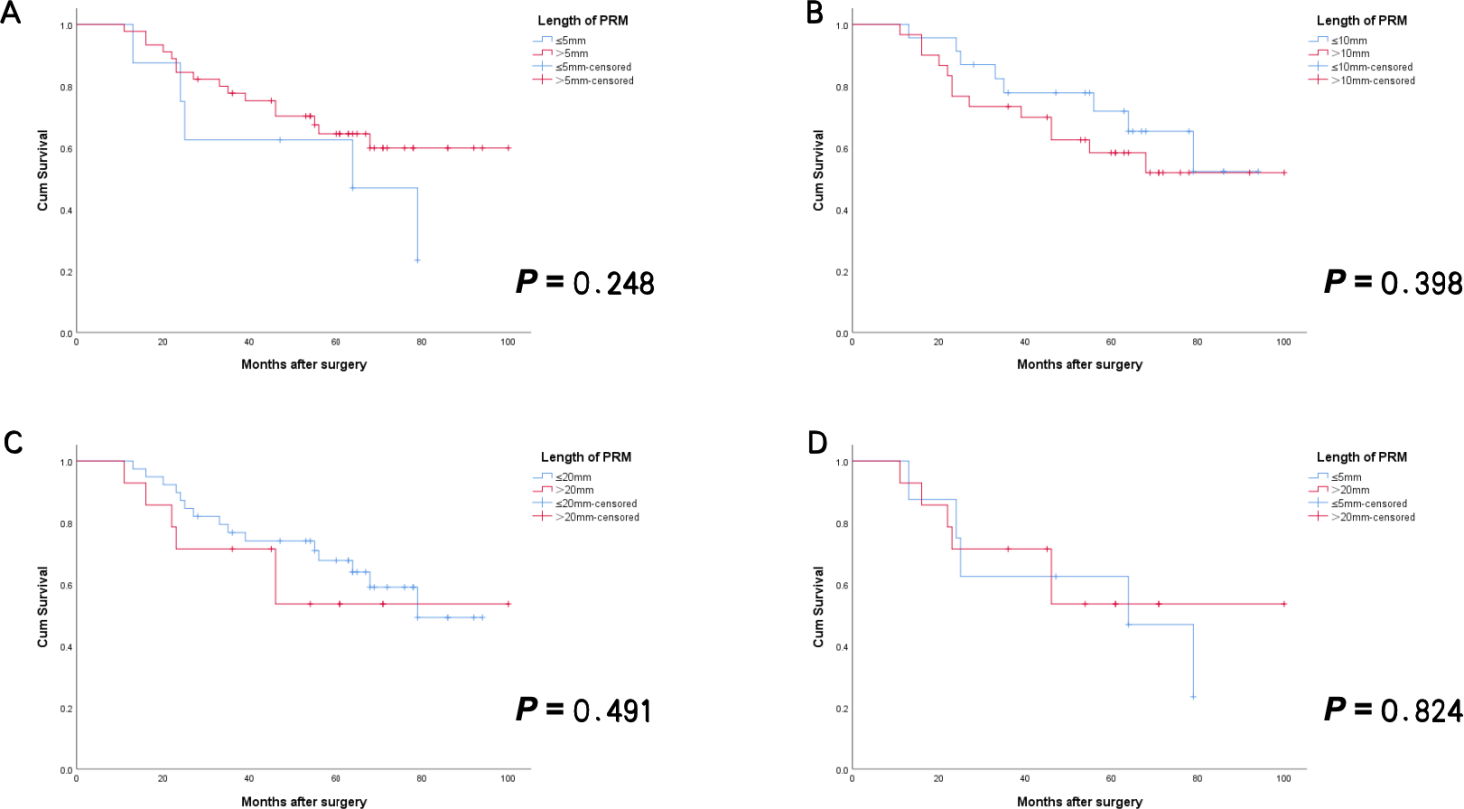
OS curves for Siewert type II AEG patients with negative PRM in NAC group. There was no statistically significant difference between patients with PRM length above and below the different cutoff values [**(A)** ≤5mm vs > 5mm; **(B)** ≤10mm vs > 10mm; **(C)** ≤20mm vs > 20mm; **(D)** ≤5mm vs > 20mm]. (All *p*>0.05)]. AEG, adenocarcinoma of esophagogastric junction; NAC, neoadjuvant chemotherapy; PRM, proximal resection margin; OS, overall survival.

Among patients in NAC group, subgroups were stratified according to the aforementioned cutoff values, and differences in clinicopathological characteristics and survival outcomes were compared. The results showed no statistically significant differences in clinicopathological characteristics, OS, or RFS among the subgroups in NAC group. Specifically, no statistically significant differences in clinicopathological features or survival outcomes were observed between patients with PRM lengths ≤5 mm and those with lengths >20 mm (**Table 6A**, **Figures 3**, **4**).

**Table 6A:** Comparison of clinicopathological characteristics and survival outcomes by patients with different length of PRM in NAC group.

Variable	Length of PRM in NAC group											
	≤5mm (n=8)	>5mm (n=45)	*P* value	≤10mm (n=23)	>10mm (n=30)	*P* value	≤20mm (n=39)	>20mm (n=14)	*P* value	≤5mm (n=8)	>20mm (n=14)	*P* value
Gender			0.116			0.597			0.108			0.163
Male	8 (100.0)	34 (75.6)		19 (82.6)	23 (76.7)		33 (84.6)	9 (64.3)		8 (100.0)	9 (64.3)	
Female	0 (0.0)	11 (24.4)		4 (17.4)	7 (23.3)		6 (15.4)	5 (35.7)		0 (0.0)	5 (35.7)	
Age	60.4±4.1	60.3±10.1	0.981	60.2±7.9	60.3±10.6	0.955	60.9±7.1	58.7±14.2	0.594	60.4±4.1	58.7±14.2	0.689
CEA			0.531			0.125			0.506			0.752
Normal	5 (62.5)	33 (73.3)		14 (60.9)	24 (80.0)		27 (69.2)	11 (78.6)		5 (62.5)	11 (78.6)	
Abnormal	3 (37.5)	12 (26.7)		9 (39.1)	6 (20.0)		12 (30.8)	3 (21.4)		3 (37.5)	3 (21.4)	
CA19_9			0.285			0.231			0.890			0.958
Normal	6 (75.0)	40 (88.9)		18 (78.3)	28 (93.3)		34 (87.2)	12 (85.7)		6 (75.0)	12 (85.7)	
Abnormal	2 (25.0)	5 (11.1)		5 (21.7)	2 (6.7)		12 (12.8)	2 (14.3)		2 (25.0)	2 (14.3)	
Borrmann type			0.38			0.195			0.601			0.726
Type 1+2	0 (0.0)	4 (8.9)		0 (0.0)	4 (13.3)		2 (5.1)	2 (14.3)		0 (0.0)	2 (14.3)	
Type 3+4	8 (100.0)	41 (91.1)		23 (100.0)	26 (86.7)		37 (94.9)	12 (85.7)		8 (100.0)	12 (85.7)	
Length of PRM	0.38±0.18	1.53±0.62	<0.001	0.74±0.31	1.83±0.54	<0.001	1.01±0.42	2.32±0.3	<0.001	0.38±0.18	2.32±0.3	<0.001
Mass size			0.532			0.597			0.108			0.497
<5 cm	7 (87.5)	35 (77.8)		19 (82.6)	23 (76.7)		33 (84.6)	9 (64.3)		7 (87.5)	9 (64.3)	
≥5 cm	1 (12.5)	10(22.2)		4 (17.4)	7 (23.3)		6 (15.4)	5 (35.7)		1 (12.5)	5 (35.7)	
Differentiation			0.577			0.523			0.104			0.269
Well	5 (62.5)	20 (44.4)		12 (52.2)	13 (43.3)		21 (53.8)	4 (28.6)		5 (62.5)	4 (28.6)	
Poor	3 (37.5)	25 (55.6)		11 (47.8)	17 (56.7)		18 (46.2)	10 (71.4)		3 (37.5)	10 (71.4)	
Lauren classification			0.435			0.909			0.442			0.612
Intestinal	6 (75.0)	26 (60.5)		14 (63.6)	18 (62.1)		25 (65.8)	7 (53.8)		6 (75.0)	7 (53.8)	
Others	2 (25.0)	17 (39.5)		8 (36.4)	11 (37.9)		13 (34.2)	6 (46.2)		2 (25.0)	6 (46.2)	
VLI			0.198			0.823			0.734			0.497
Negative	7 (87.5)	29 (64.4)		16 (69.6)	20 (66.7)		27 (69.2)	9 (64.3)		7 (87.5)	9 (64.3)	
Positive	1 (12.5)	16 (35.6)		7 (30.4)	10 (33.3)		12 (30.8)	5 (35.7)		1 (12.5)	5 (35.7)	
Perineural invasion			0.577			0.933			0.805			0.903
Negative	3 (37.5)	25 (55.6)		12 (52.2)	16 (53.3)		21 (53.8)	7 (50.0)		3 (37.5)	7 (50.0)	
Positive	5 (62.5)	20 (44.4)		11 (47.8)	14 (46.7)		18 (46.2)	7 (50.0)		5 (62.5)	7 (50.0)	
pT			0.748			0.402			0.487			0.958
pT0-2	2 (25.0)	9 (20.0)		6 (26.1)	5 (16.7)		9 (23.1)	2 (14.3)		2 (25.0)	2 (14.3)	
pT3-4	6 (75.0)	36 (80.0)		17 (73.9)	25 (83.3)		30 (76.9)	12 (85.7)		6 (75.0)	12 (85.7)	
pN			0.748			0.877			0.942			0.848
pN0-1	6 (75.0)	36 (80.0)		18 (78.3)	24 (80.0)		31 (79.5)	11 (78.6)		6 (75.0)	11 (78.6)	
pN2-3	2 (25.0)	9 (20.0)		5 (21.7)	6 (20.0)		8 (20.5)	3 (21.4)		2 (25.0)	3 (21.4)	
pTNM			0.973			0.679			0.257			0.604
pTNM0-2	6 (75.0)	34 (75.6)		18 (78.3)	22 (73.3)		31 (79.5)	9 (64.3)		6 (75.0)	9 (64.3)	
pTNM3	2 (25.0)	11 (24.4)		5 (21.7)	8 (26.7)		8 (20.5)	5 (35.7)		2 (25.0)	5 (35.7)	
5 Y Survival rate (%)	5 (62.5)	30 (66.7)	0.819	17 (73.9)	18 (60.0)	0.289	27 (69.2)	8 (57.1)	0.413	5 (62.5)	8 (57.1)	0.806
5 Y Recurrence-free rate (%)	5 (62.5)	28 (62.2)	0.988	17 (73.9)	16 (53.3)	0.126	25 (64.1)	8 (57.1)	0.645	5 (62.5)	8 (57.1)	0.806
OS (m)	54.8±10.8	74.5±5.1	0.248	72.8±6.1	68.6±6.6	0.398	69.9±4.9	66.9±10.2	0.491	54.8±10.8	66.9±10.2	0.824
RFS (m)	51.5±12.4	67.1±5.8	0.405	68.5±6.7	60.4±7.3	0.297	62.5±5.6	65.8±10.7	0.908	51.5±12.4	65.8±10.7	0.684

PRM, proximal resection margin; NAC, Neoadjuvant chemotherapy; CEA, carcinoembryonic antigen; CA19_9, carbohydrate antigen 19-9; VLI, Vascular-lymphatic invasion; pTNM, pathologic tumor-node-metastasis staging; OS, Overall survival; RFS, Recurrence-free survival.

*PRM length was analyzed as a categorical variable.

**Table 6B:** Comparison of clinicopathological characteristics and survival outcomes by patients with different length of PRM in SA group.

Variable	Length of PRM in SA group											
	≤5mm (n=8)	>5mm (n=45)	*P* value	≤10mm (n=23)	>10mm (n=30)	*P* value	≤20mm (n=39)	>20mm (n=14)	*P* value	≤5mm (n=8)	>20mm (n=14)	*P* value
Gender			0.275			0.487			0.663			0.315
Male	12 (92.3)	30 (78.9)		24 (85.7)	18 (78.3)		31 (83.8)	11 (78.6)		12 (92.3)	11 (78.6)	
Female	1 (7.7)	8 (21.1)		4 (14.3)	5 (21.7)		6 (16.2)	3 (21.4)		1 (7.7)	3 (21.4)	
Age	61.5±10.0	61.5±12.0	0.997	62.4±9.4	60.3±13.6	0.505	62.2±9.5	59.6±15.8	0.571	61.5±10.0	59.6±15.8	0.716
CEA			0.373			0.232			0.052			0.449
Normal	10 (83.3)	26 (70.3)		18 (66.7)	18 (81.8)		23 (65.7)	13 (92.9)		10 (83.3)	13 (92.9)	
Abnormal	2 (16.7)	11 (29.7)		9 (33.3)	4 (18.2)		12 (34.3)	1 (7.1)		2 (16.7)	1 (7.1)	
CA19_9			0.861			0.477			0.641			0.867
Normal	10 (83.3)	30 (81.1)		23 (85.2)	17 (77.3)		28 (80.0)	12 (85.7)		10 (83.3)	12 (85.7)	
Abnormal	2 (16.7)	7 (18.9)		4 (14.8)	5 (22.7)		7 (20.0)	2 (14.3)		2 (16.7)	2 (14.3)	
Borrmann type			0.464			0.779			0.943			0.586
Type 1+2	1 (7.7)	6 (15.8)		3 (10.7)	4 (17.4)		5 (13.5)	2 (14.3)		1 (7.7)	2 (14.3)	
Type 3+4	12 (92.3)	32 (84.2)		25 (89.3)	19 (82.6)		32 (86.5)	12 (85.7)		12 (92.3)	12 (85.7)	
Length of PRM	0.35±0.19	1.54±0.81	<0.001	0.61±0.30	2.00±0.73	<0.001	0.81±0.47	2.36±0.69	<0.001	0.35±0.19	2.36±0.69	<0.001
Mass size			0.168			0.056			0.145			0.085
<5 cm	5 (38.5)	23 (60.5)		12 (42.9)	16 (69.6)		18 (48.6)	10 (71.4)		5 (38.5)	10 (71.4)	
≥5 cm	8 (61.5)	15 (39.5)		16 (57.1)	7 (30.4)		19 (51.4)	4 (28.6)		8 (61.5)	4 (28.6)	
Differentiation			0.045			0.304			0.104			0.746
Well	3 (23.1)	21 (55.3)		15 (53.6)	9 (39.1)		20 (54.1)	4 (28.6)		3 (23.1)	4 (28.6)	
Poor	10 (76.9)	17 (44.7)		13 (46.4)	14 (60.9)		17 (45.9)	10 (71.4)		10 (76.9)	10 (71.4)	
Lauren classification			0.366			0.238			0.061			0.581
Intestinal	6 (46.2)	23 (60.5)		18 (64.3)	11 (47.8)		24 (64.9)	5 (35.7)		6 (46.2)	5 (35.7)	
Others	7 (53.8)	15 (39.5)		10 (35.7)	12 (52.2)		13 (35.1)	9 (64.3)		7 (53.8)	9 (64.3)	
VLI			0.901			0.276			0.032			0.114
Negative	4 (30.8)	11 (28.9)		10 (35.7)	5 (21.7)		14 (37.8)	1 (7.1)		4 (30.8)	1 (7.1)	
Positive	9 (69.2)	27 (71.1)		18 (64.3)	18 (78.3)		23 (62.2)	13 (92.9)		9 (69.2)	13 (92.9)	
Perineural invasion			0.214			0.229			0.259			0.152
Negative	5 (38.5)	8 (21.1)		9 (32.1)	4 (17.4)		11 (29.7)	2 (14.3)		5 (38.5)	2 (14.3)	
Positive	8 (61.5)	30 (78.9)		19 (67.9)	19 (82.6)		26 (70.3)	12 (85.7)		8 (61.5)	12 (85.7)	
pT			0.555			0.921			0.101			0.326
pT0-2	0 (0.0)	1 (2.6)		0 (0.0)	1 (4.3)		0 (0.0)	1 (7.1)		0 (0.0)	1 (7.1)	
pT3-4	13 (100.0)	37 (97.4)		28 (100.0)	22 (95.7)		37 (100.0)	13 (92.9)		13 (100.0)	13 (92.9)	
pN			0.957			0.896			0.277			0.516
pN0-1	4 (30.8)	12 (31.6)		9 (32.1)	7 (30.4)		10 (27.0)	6 (42.9)		4 (30.8)	6 (42.9)	
pN2-3	9 (69.2)	26 (68.4)		19 (67.9)	16 (69.6)		27 (73.0)	8 (57.1)		9 (69.2)	8 (57.1)	
pTNM			0.423			0.696			0.207			0.228
pTNM0-2	2 (15.4)	10 (26.3)		6 (21.4)	6 (26.1)		7 (18.9)	5 (35.7)		2 (15.4)	5 (35.7)	
pTNM3	11 (84.6)	28 (73.7)		22 (78.6)	17 (73.9)		30 (81.1)	9 (64.3)		11 (84.6)	9 (64.3)	
5 Y Survival rate (%)	7 (53.8)	21 (55.3)	0.929	15 (53.6)	13 (56.5)	0.833	19 (51.4)	9 (64.3)	0.407	7 (53.8)	9 (64.3)	0.581
5 Y Recurrence-free rate (%)	5 (38.5)	17 (44.7)	0.693	10 (35.7)	12 (52.2)	0.238	14 (37.8)	8 (57.1)	0.214	5 (38.5)	8 (57.1)	0.332
OS (m)	69.7±11.1	63.2±5.8	0.678	65.2±7.6	66.6±7.2	0.832	66.0±6.4	67.3±9.8	0.778	69.7±11.1	67.3±9.8	0.954
PFS (m)	52.2±11.0	55.7±6.5	0.783	48.6±7.4	62.4±8.1	0.234	51.6±6.4	63.3±10.9	0.356	52.2±11.0	63.3±10.9	0.468

PRM, proximal resection margin; SA, Surgery alone; CEA, carcinoembryonic antigen; CA19_9, carbohydrate antigen 19-9; VLI, Vascular-lymphatic invasion; pTNM, pathologic tumor-node-metastasis staging; OS, Overall survival; RFS, Recurrence-free survival. *PRM length was analyzed as a categorical variable.

**Figure 5:**
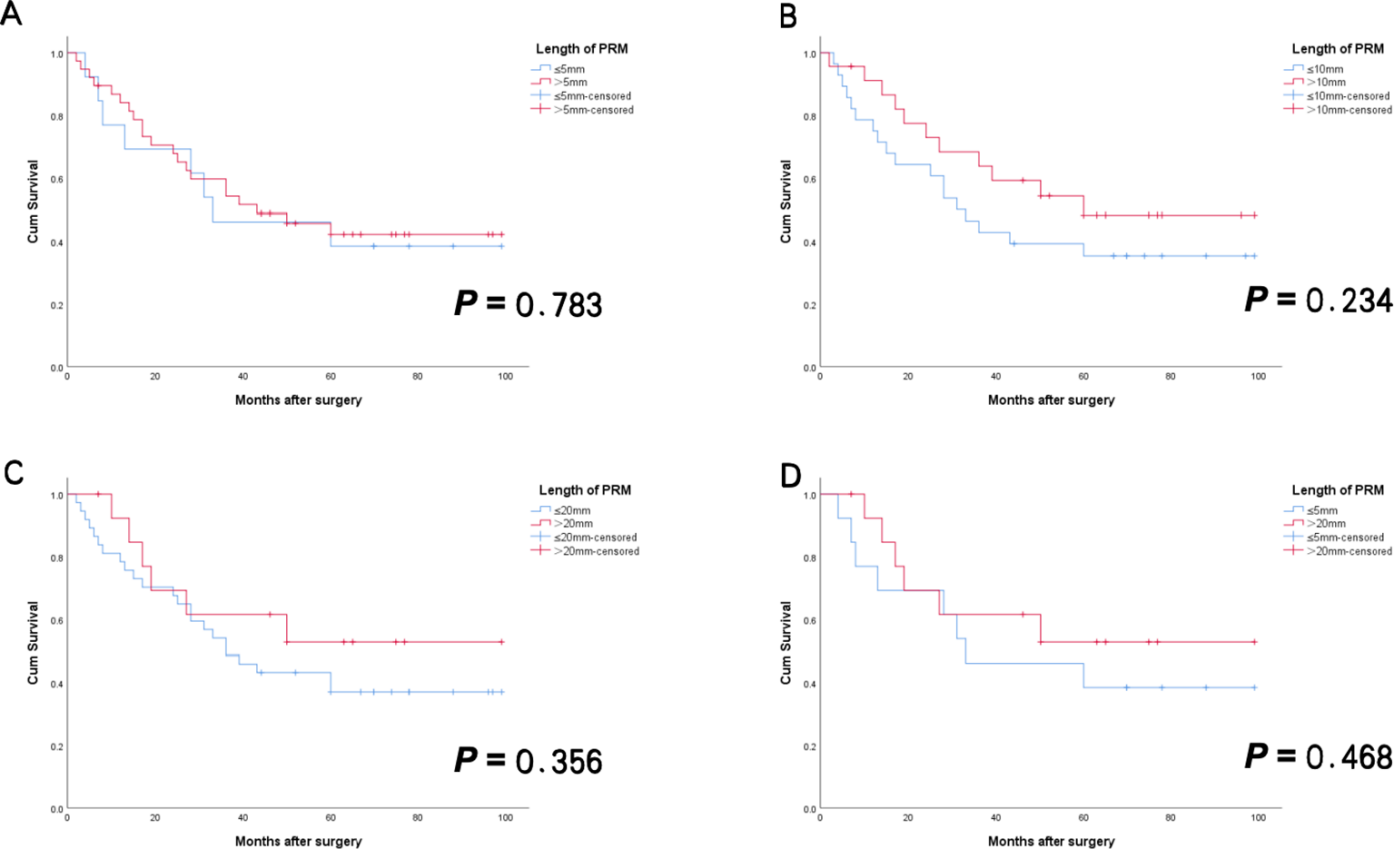
RFS curves for Siewert type II AEG patients with negative PRM in SA group. There was no statistically significant difference between patients with PRM length above and below the different cutoff values [**(A)** ≤5mm vs > 5mm; **(B)** ≤10mm vs > 10mm; **(C)** ≤20mm vs > 20mm; **(D)** ≤5mm vs > 20mm]. (All *p*>0.05)]. AEG, adenocarcinoma of esophagogastric junction; SA, surgery alone; PRM, proximal resection margin; RFS, recurrence-free survival.

**Figure 6:**
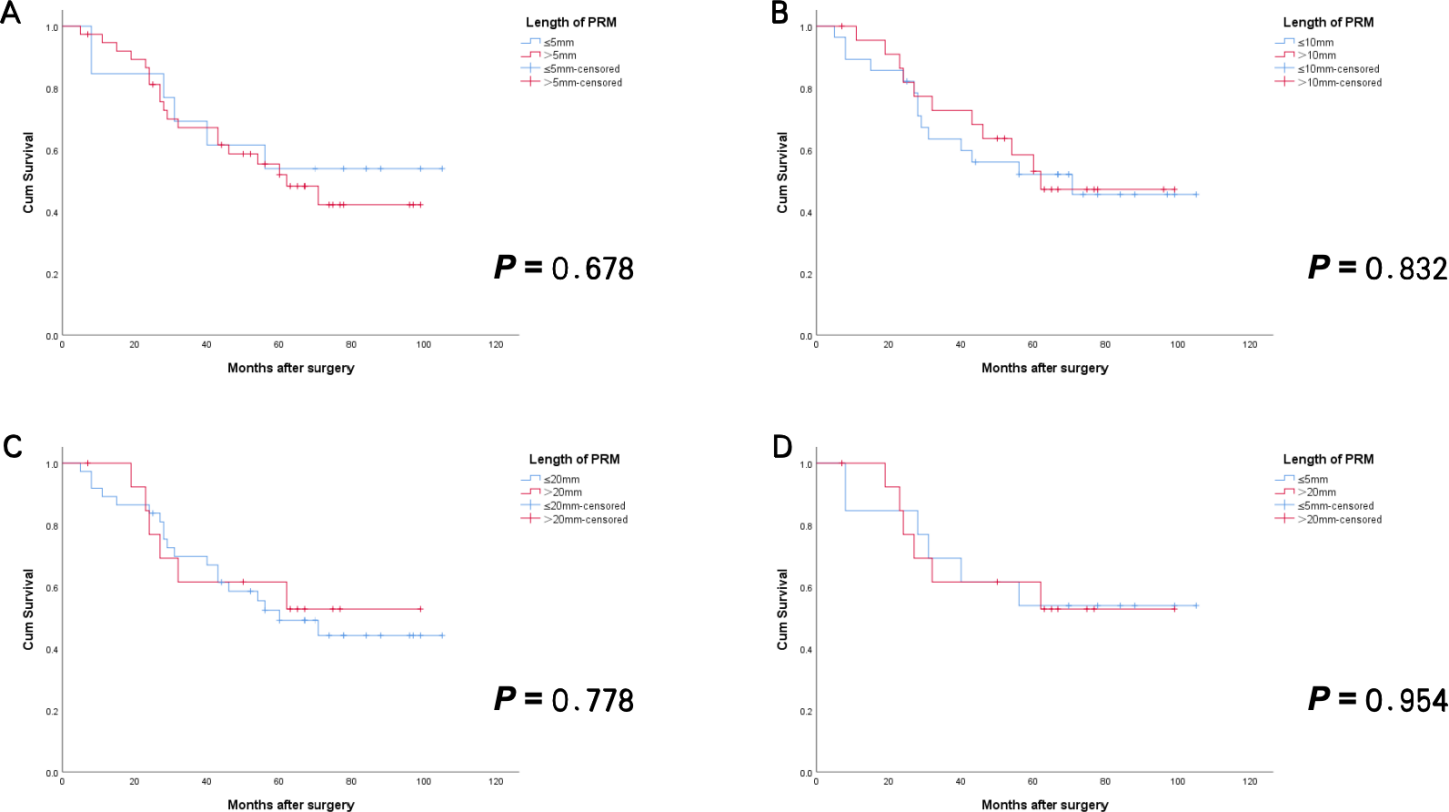
OS curves for Siewert type II AEG patients with negative PRM in SA group. There was no statistically significant difference between patients with PRM length above and below the different cutoff values [ **(A)** ≤5mm vs > 5mm; **(B)** ≤10mm vs > 10mm; **(C)** ≤20mm vs > 20mm; **(D)** ≤5mm vs > 20mm]. (All *p*>0.05)]. AEG, adenocarcinoma of esophagogastric junction; SA, surgery alone; PRM, proximal resection margin; OS, overall survival.

Using the same methodology, subgroup comparisons were carried out among patients in SA group to evaluate differences in clinicopathological characteristics and survival outcomes. The results indicated that, apart from a few comparisons where statistically significant differences in certain clinicopathological features were observed—such as a significantly lower rate of well-differentiated tumors in the ≤5 mm group compared to the >5 mm group (*p* = 0.045), and a significantly lower rate of lymph-vascular invasion in the ≤20 mm group compared to the >20 mm group (*p* = 0.032)—the majority of clinicopathological characteristics showed no statistically significant differences. Furthermore, no statistically significant differences in OS or RFS were found among the subgroups in SA group (**Table 6B**, **Figures 5**, **6**).

In patients with negative PRM status, subgroups were classified using 5 mm, 10 mm, and 20 mm as cut-off values. Clinicopathological characteristics and survival outcomes were compared across different treatment strategies within each subgroup. The results showed there were no statistically significant differences in RFS or OS among patients receiving different treatment strategies in any of the subgroups (**Table 7**).

**Table 7:** Comparison of clinicopathological characteristics and survival outcomes by patients in SA group and NAC group with different length of PRM.

Variable	Length of PRM≤5mm			Length of PRM>5mm			Length of PRM ≤10mm			Length of PRM >10mm			Length of PRM ≤20mm			Length of PRM >20mm		
	SA (n=13)	NAC (n=8)	*P* value	SA (n=38)	NAC (n=45)	*P* value	SA (n=28)	NAC (n=23)	*P* value	SA (n=23)	NAC (n=30)	*P* value	SA (n=37)	NAC (n=39)	*P* value	SA (n=14)	NAC (n=14)	*P* value
Gender			0.421			0.714			0.762			0.891			0.921			0.676
Male	12 (92.3)	8 (100.0)		30 (78.9)	34 (75.6)		24 (85.7)	19 (82.6)		18 (78.3)	23 (76.7)		31 (83.8)	33 (84.6)		11 (78.6)	9 (64.3)	
Female	1 (7.7)	0 (0.0)		8 (21.1)	11 (24.4)		4 (14.3)	4 (17.4)		5 (21.7)	7 (23.3)		6 (16.2)	6 (15.4)		3 (21.4)	5 (35.7)	
Age	61.5±10.0	60.4±4.1	0.734	61.5±12.0	60.3±10.1	0.634	62.4±9.4	60.2±7.9	0.375	60.3±13.6	60.4±10.6	0.975	62.2±9.5	60.9±7.1	0.506	59.6±15.8	58.7±14.2	0.881
CEA			0.598			0.759			0.670			0.869			0.747			0.589
Normal	10 (83.3)	5 (62.5)		26 (70.3)	33 (73.3)		18 (66.7)	14 (60.9)		18 (81.8)	24 (80.0)		23 (65.7)	27 (69.2)		13 (92.9)	11 (78.6)	
Abnormal	2 (16.7)	3 (37.5)		11 (29.7)	12 (26.7)		9 (33.3)	9 (39.1)		14 (18.2)	6 (20.0)		12 (34.3)	12 (30.8)		1 (7.1)	3 (21.4)	
CA19_9			0.648			0.320			0.790			0.206			0.403			1.000
Normal	10 (83.3)	6 (75.0)		30 (81.1)	40 (88.9)		23 (85.2)	18 (78.3)		17 (77.3)	28 (93.3)		28 (80.0)	34 (87.2)		12 (85.7)	12 (85.7)	
Abnormal	2 (16.7)	2 (25.0)		7 (18.9)	5 (11.1)		4 (14.8)	5 (21.7)		5 (22.7)	2 (6.7)		7 (20.0)	5 (12.8)		2 (14.3)	2 (14.3)	
Borrmann type			0.421			0.336			0.308			0.683			0.386			1.000
Type 1+2	1 (7.7)	0 (0.0)		6 (15.8)	4 (8.9)		3 (10.7)	0 (0.0)		4 (17.4)	4 (13.3)		5 (13.5)	2 (5.1)		2 (14.3)	2 (14.3)	
Type 3+4	12 (92.3)	8 (100.0)		32 (84.2)	41 (91.1)		25 (89.3)	23 (100.0)		19 (82.6)	26 (86.7)		32 (86.5)	37 (94.9)		12 (85.7)	12 (85.7)	
Length of PRM	0.35±0.19	0.38±0.18	0.732	1.54±0.81	1.53±0.62	0.958	0.61±0.30	0.73±0.31	0.157	2.00±0.73	1.83±0.54	0.355	0.81±0.47	1.01±0.42	0.054	2.36±0.69	2.32±0.37	0.866
Mass size			0.080			0.088			0.004			0.561			0.001			0.686
<5 cm	5 (38.5)	7 (87.5)		23 (60.5)	36 (77.8)		12 (42.9)	19 (82.6)		16 (69.6)	23 (76.7)		18 (48.6)	33 (84.6)		10 (71.4)	9 (64.3)	
≥5 cm	8 (61.5)	1 (12.5)		15 (39.5)	10 (22.2)		16 (57.1)	4 (17.4)		7 (30.4)	7 (23.3)		19 (51.4)	6 (15.4)		4 (28.4)	5 (35.7)	
Differentiation			0.179			0.326			0.921			0.758			0.985			1
Well	3 (23.1)	5 (62.5)		21 (55.3)	20 (44.4)		15 (53.6)	12 (52.2)		9 (39.1)	13 (43.3)		20 (54.1)	21 (53.8)		4 (28.4)	4 (28.4)	
Poor	10 (76.9)	3 (37.5)		17 (44.7)	25 (55.6)		13 (46.4)	11 (47.8)		14 (60.9)	17 (56.7)		17 (45.9)	18 (46.2)		10 (71.4)	10 (71.4)	
Lauren classification			0.399			0.996			0.962			0.304			0.933			0.343
Intestinal	6 (46.2)	6 (75.0)		23 (60.5)	26 (60.5)		18 (64.3)	14 (63.6)		11 (47.8)	18 (62.1)		24 (64.9)	25 (65.8)		5 (35.7)	7 (53.8)	
Others	7 (53.8)	2 (25.0)		15 (39.5)	17 (39.5)		10 (35.7)	8 (36.4)		12 (52.2)	11 (37.9)		13 (35.1)	13 (34.2)		9 (64.3)	6 (46.2)	
VLI			0.038			0.001			0.016			0.001			0.006			0.002
Negative	4 (30.8)	7 (87.5)		11 (28.9)	29 (64.4)		10 (35.7)	16 (69.6)		5 (21.7)	20 (66.7)		14 (37.8)	27 (69.2)		1 (7.1)	9 (64.3)	
Positive	9 (69.2)	1 (12.5)		27 (71.1)	16 (35.6)		18 (64.3)	7 (30.4)		18 (78.3)	10 (33.3)		23 (62.2)	12 (30.8)		13 (92.9)	5 (35.7)	
Perineural invasion			0.965			0.001			0.148			0.007			0.033			0.106
Negative	5 (38.5)	3 (37.5)		8 (21.1)	25 (55.6)		9 (32.1)	12 (52.2)		4 (17.4)	16 (53.3)		11 (29.7)	21 (53.8)		2 (14.3)	7 (50.0)	
Positive	8 (61.5)	5 (62.5)		30 (78.9)	20 (44.4)		19 (67.9)	11 (47.8)		19 (82.6)	14 (46.7)		26 (70.3)	18 (46.2)		12 (85.7)	7 (50.0)	
pT			0.259			0.015			0.015			0.334			0.006			0.541
pT0-2	0 (0.0)	2 (25.0)		1 (2.6)	9 (20.0)		0 (0.0)	6 (26.1)		1 (4.3)	5 (16.7)		0 (0.0)	9 (23.1)		1 (7.1)	2 (14.3)	
pT3-4	13 (100.0)	6 (75.0)		37 (97.4)	36 (80.0)		28 (100.0)	17 (73.9)		22 (95.7)	25 (83.3)		37 (100.0)	30 (76.9)		13 (92.9)	12 (85.7)	
pN			0.128			<0.001			0.001			<0.001			<0.001			0.053
pN0-1	4 (30.8)	6 (75.0)		12 (31.6)	36 (80.0)		9 (32.1)	18 (78.3)		7 (30.4)	24 (80.0)		10 (27.0)	31 (79.5)		6 (42.9)	11 (78.6)	
pN2-3	9 (69.2)	2 (25.0)		26 (68.4)	9 (20.0)		19 (67.9)	5 (21.7)		16 (69.6)	6 (20.0)		27 (73.0)	8 (20.5)		8 (57.1)	3 (21.4)	
pTNM			0.023			<0.001			<0.001			0.001			<0.001			0.131
pTNM0-2	2 (15.4)	6 (75.0)		10 (26.4)	34 (75.6)		6 (21.4)	18 (78.3)		6 (26.1)	22 (73.3)		7 (18.9)	31 (79.5)		5 (35.7)	9 (64.3)	
pTNM3	11 (84.6)	2 (25.0)		28 (73.7)	1 (24.4)		22 (78.6)	5 (21.7)		17 (73.9)	8 (26.7)		30 (81.1)	8 (20.5)		9 (64.3)	5 (35.7)	
5 Y Survival rate (%)	7 (53.8)	5 (62.5)	0.697	21 (55.3)	30 (66.7)	0.288	15 (53.6)	17 (73.9)	0.135	13 (56.5)	18 (60.0)	0.799	19 (51.4)	27 (69.2)	0.111	9 (64.3)	8 (57.1)	0.699
5 Y Recurrence-free rate (%)	5 (38.5)	5 (62.5)	0.284	17 (44.7)	28 (62.2)	0.111	10 (35.7)	17 (73.9)	0.007	12 (52.2)	16 (53.3)	0.933	14 (37.8)	25 (64.1)	0.022	8 (57.1)	8 (57.1)	1.000
OS (m)	69.7±11.1	54.8±10.8	0.510	63.2±5.8	74.5±5.1	0.171	65.2±7.6	72.8±6.1	0.262	66.6±7.1	68.6±6.6	0.797	66.0±6.4	69.9±4.9	0.273	67.3±9.8	66.9±10.2	0.887
PFS (m)	52.2±11.0	51.5±12.4	0.903	55.7±6.5	67.1±5.8	0.188	48.6±7.4	68.5±6.7	0.054	62.4±8.1	60.4±7.3	0.876	51.6±6.4	62.5±5.6	0.131	63.3±10.9	65.8±10.7	0.954

SA, Surgery alone; NAC, neoadjuvant chemotherapy; PRM, proximal resection margin; CEA, carcinoembryonic antigen; CA19_9, carbohydrate antigen 19-9; VLI, Vascular-lymphatic invasion; pTNM, pathologic tumor-node-metastasis staging; OS, Overall survival; RFS, Recurrence-free survival.

*PRM length was analyzed as a categorical variable.

## 4 Discussion

To determine the optimal PRM length for AEG, two viewpoints had historically emerged (26). One advocated that the length should ensure pathological negativity to the greatest extent possible; the other, from a prognostic perspective, employed statistical methods to identify the optimal PRM length associated with the highest survival rate. A significant discrepancy was observed between the two viewpoints: the former generally considered a length of 6–8 cm (or more) necessary to achieve pathological negativity in all patients (2, 4, 5), whereas the latter revealed that a length exceeding 2 cm yield significantly better survival outcomes than shorter length, thus advocating 2 cm was sufficient (22).

Upon closer analysis, both viewpoints presented certain limitations. In the first case, the required length of PRM was determined by a few extreme cases in the cohort – patients with the farthest spread of the tumor through mucosa and/or submucosa or skipping metastasis. Applying the length obtained from extreme cases as universal standard would necessitate extensive distal esophageal resection in all patients, inevitably increasing the rate of thoracotomies (2, 10, 27) and corresponding perioperative risks (11). Moreover, opponents noted that patients with skip metastases often presented with extensive lymphatic invasion; and their prognosis exhibited poor even with additional esophageal resection (28). Meanwhile, the second viewpoint underestimated the importance of the PRM status. While it might achieve the best overall survival at the population level, individual patient could still have opportunities for positive PRM and then suffer high postoperative recurrence rates and poor survival outcomes.

Therefore, this study explores a novel therapeutic strategy that prioritizes achieving negative status of PRM before investigating the relationship between the length and survival outcomes. We propose that this approach avoids the rigidity of a one-size-fits-all method while also circumventing the limitation of solely focusing on survival optimization in large cohorts at the expense of individual outcomes. In summary, this strategy emphasizes both the importance of negative status of PRM and the consideration of survival impact. Although this method has been reported in gastric cancer research (24, 25), it is less documented in AEG (19) and has not been reported in patients with AEG following NAC.

In this study, we found that patients with positive PRM had significantly more advanced pathological stages compared to those with negative status, and the difference was statistically significant. However, no statistically significant differences were observed in various clinicopathological characteristics or the length of PRM between the two groups. This suggested that a positive PRM was not related to surgical technique or quality but might be associated with stage and adverse biological behavior (29–32). Survival analysis indicated that patients with positive PRM had worse OS and RFS than those with negative margins, with the difference in RFS being statistically significant. These findings were consistent with reports in the literature from 2021 (31). Multivariate analysis further confirmed that PRM status was an independent risk factor for RFS in patients with Siewert type II AEG.

Given that the status of the PRM was associated with survival outcomes and prognosis, we excluded patients with positive PRM in the subsequent analysis, focusing specifically on those of locally advanced Siewert type II AEG with negative PRM. In this cohort, patients were divided into two groups based on the treatment strategy received: SA group and NAC group. In the SA group, survival analysis indicated that PRM length was not an independent factor influencing patients’ survival or prognosis, which was consistent with the findings of Feng et al. (19). A more detailed comparison revealed that, in our study, the patients in SA group had a TNM stage III rate of 76.5%, a PRM length of 1.24 ± 0.88 cm, and a 5-year overall survival rate of 54.9%. In contrast, Feng et al. reported a stage III rate of 54.8%, a proximal margin length of 2.24 ± 0.99 cm, and a comparable survival rate (54.7%). Thus, despite differences in PRM length, the two cohorts achieved similar survival outcomes, supporting the conclusion that PRM length was not associated with survival. To further strengthen the reliability of this conclusion, we referred to previous studies and evaluated different cutoff values for PRM length, comparing survival outcomes above and below these thresholds (25). The results showed that regardless of whether the cutoff was set at 5 mm, 10 mm, or 20 mm, there were no statistically significant differences in OS or RFS between the subgroups. In particular, even when comparing patients with PRM lengths ≤5 mm and those with lengths >20 mm, no statistically significant survival difference was observed. Therefore, it could conclude that in SA group with negative status PRM, PRM length was not associated with patient survival.

In addition to confirming that the PRM length was not associated with survival outcomes in locally advanced AEG patients with negative PRM in the SA group, this study further investigated the relationship between PRM length and survival prognosis in patients who underwent NAC followed surgery. The latter held significant clinical relevance for two main reasons: firstly, previous studies rarely addressed the impact of PRM length on prognosis in patients receiving NAC (19, 20); secondly, in these patients, tumors might exhibit varying degrees of regression. Consequently, determining tumor boundaries and PRM length after NAC posed greater challenges compared to surgical resection alone in patients with locally advanced AEG. To address this, we employed techniques such as visual inspection, palpation, and intraoperative gastroscopy (33) to generally determine the upper boundary of the tumor. Furthermore, considering tumor regression after chemotherapy, we precisely measured the PRM length by examining formalin-fixed specimens microscopically.

In this cohort, 53 patients (49%) received NAC. Comparative analysis of clinicopathological characteristics revealed that the NAC group had significantly smaller tumor size, lower rates of lymph-vascular invasion and perineural invasion, as well as lower pTNM staging compared to the SA group. These findings indicated that NAC could reduce tumor size, mitigate adverse histologic features, and achieve downstaging—results consistent with previous studies (34). However, although tumor size decreased in the NAC group, there was no statistically significant difference in PRM length between the two groups. This suggested that the reduction in tumor diameter after NAC did not occur along the longitudinal axis and therefore did not lead to a significant change in PRM length. Additionally, although RFS and OS were longer in the NAC group than in the SA group, the differences were not statistically significant. This was also consistent with some prior researches indicating that while NAC might improve resectability, it did not significantly prolong patient survival (35, 36). On the other side, the survival comparisons between NAC and SA groups in this study were descriptive and not intended to demonstrate treatment efficacy.

In patients receiving NAC, results revealed that PRM length was not an independent factor influencing survival or prognosis. Similarly, different cutoff values were applied to categorize PRM lengths, and survival outcomes were compared across subgroups. No statistically significant differences were observed in survival outcomes. Furthermore, a comparison of clinicopathological characteristics and survival between the two extreme subgroups—PRM length ≤5 mm and >20 mm—also showed no statistically significant differences. These findings indicated that among patients who had undergone NAC, as long as the status of PRM was negative, survival outcomes were not associated with the length of PRM.

This study indicated that the length of PRM was not associated with survival prognosis, provided that the status of PRM was negative. For AEG, submucosal infiltration may occur, making visual assessment of normal mucosa alone insufficient to determine a negative PRM. Particularly after NAC, tumor regression may render visual mucosal evaluation even less reliable for assessing margin status. Intraoperative FSA provided critical information on margin involvement. For patients with positive result of FSA, extended resection to achieve negative PRM offered the opportunity for R0 resection, which might improve postoperative survival (37, 38). However, previous studies have shown that even after conversion to negative status, survival in locally advanced patients does not improve (39), leading to doubts regarding the value of FSA.

Until 2025, an Indian study (40) demonstrated that survival improved after achieving negative margins, including in locally advanced Siewert type II AEG patients, thereby reaffirming the value of intraoperative FSA. In present study, five patients with PRM involved on FSA underwent re-resection intraoperatively, all achieving negative margins (R1-to-R0 group). Survival outcomes were compared among R1-to-R0 group, those with positive margins on histological pathology (R1 group), and those with negative margins (R0 group). Although limited by small sample size, this exploratory analysis showed that patients in R1-to-R0 group had significantly better survival than R1 group (RFS: 65.6 ± 14.9 vs. 16.0 ± 4.3, p=0.025; OS: 67.6 ± 14.0 vs. 46.0 ± 11.5, p=0.264) and had no statistically significant difference compared to R0 group (RFS: 65.6 ± 14.9 vs. 59.6 ± 4.0, p=0.595; OS: 67.6 ± 14.0 vs. 70.3 ± 3.9, p=0.814). These findings are consistent with emerging evidence from recent studies, suggesting that intraoperative margin revision may offer survival benefits. Therefore, our findings support the routine use of intraoperative FSA, which is consistent with current literature recommendations.

Our results contradicted the conclusions of Lee’s study (6). Lee observed and analyzed 29 patients with locally advanced Siewert type II AEG who underwent transabdominal total gastrectomy and D2 lymphadenectomy, with a PRM length of 7.5 ± 6.6 mm. During the follow-up period, the local recurrence rate in this group was higher than that in patients with Siewert type III AEG and upper third gastric cancer. Therefore, the author inferred that extending the PRM length may reduce the local recurrence rate. A careful analysis revealed several issues about Lee’s study: First, the sample size of locally advanced Siewert type II AEG was relatively small, compromising the reliability of its conclusions. Second, patients with Siewert type II and III, as well as upper third gastric cancer, belonged to different etiological categories, and the factors related to local recurrence were complex and could not be simply attributed to insufficient PRM length. Third, Lee’s study lacked data to support its conclusions and did not specify an optimal PRM length. Fourth, half of the AEG patients with local recurrence in Lee’s study also had distant metastases, which significantly impacted overall survival. In summary, we believed that Lee’s conclusions required further validation. Meanwhile, compared to extending the PRM length in locally advanced patients with negative PRM status, we considered it more important and feasible to select appropriate treatment strategies for patients, such as perioperative systemic therapy. In our study, the survival prognosis of patients in the NAC group was superior to that of the SA group, although the difference was not statistically significant, it still suggested a trend of improved patient outcomes with perioperative systemic therapy.

The present study has some limitations. Firstly, the retrospective design and relatively small samples would introduce selection bias. Furthermore, only 4 patients (3.7%) had positive PRM status, which resulted in wide confidence intervals and potential instability of hazard ratio estimates. Our findings regarding the prognostic impact of PRM status should be interpreted as hypothesis-generating rather than definitive conclusions. Secondly, all patients were treated at a single Chinese center with CapeOX/SOX regimens. Previous studies have suggested potential differences in tumor biology and treatment response between Eastern and Western populations. Whether our findings can be generalized to patients treated with different regimens (e.g., FLOT, ECF, et al.) warrants further investigation. Thirdly, with the advanced in clinical oncology, neoadjuvant therapy no longer consists solely of chemotherapy, but also includes neoadjuvant chemoradiotherapy, immunotherapy, and targeted therapy. The impact of these newer modalities on tumor regression patterns and optimal margin length may differ from traditional chemotherapy. Future studies should explore PRM length-prognosis relationship in patients with locally advanced Siewert type II AEG receiving these emerging therapies.

## 5 Conclusion

A positive status of PRM appears to be associated with adverse survival outcomes of patients with locally advanced Siewert type II AEG after surgery. However, for patients with negative status, the length of the PRM does not influence survival, regardless of whether they undergo surgical resection alone or NAC followed by surgery.

## Data Availability

The raw data supporting the conclusions of this article will be made available by the authors, without undue reservation.

## References

[1] Steevens J Botterweck AA Dirx MJ van der Brandt PA Schouten LJ . Trends in incidence of oesophageal and stomach cancer subtypes in Europe. Eur J Gastroenterol Hepatol. (2010) 22:669–78. doi: 10.1097/MEG.0b013e32832ca091, PMID: 19474750

[2] Mariette C Castel B Balon JM Van Seuningen I Triboulet JP . Extent of oesophageal resection for adenocarcinoma of the oesophagogastric junction. Eur J Surg Oncol. (2003) 29:588–93. doi: 10.1016/s0748-7983(03)00109-4, PMID: 12943624

[3] Mattioli S Di Simone MP Ferruzzi L D’Ovidio F Pilotti V Carella R . Surgical therapy for adenocarcinoma of the cardia: modalities of recurrence and extension of resection. Dis Esophagus. (2001) 14:104–9. doi: 10.1046/j.1442-2050.2001.00165.x, PMID: 11553218

[4] Ito H Clancy TE Osteen RT Swanson RS Bueno R Sugarbaker DJ . Adenocarcinoma of the gastric cardia: what is the optimal surgical approach? J Am Coll Surg. (2004) 199:880–6. doi: 10.1016/j.jamcollsurg.2004.08.015, PMID: 15555971

[5] Papachristou DN Agnanti N D’Agostino H Fortner JG . Histologically positive esophageal margin in the surgical treatment of gastric cancer. Am J Surg. (1980) 139:711–3. doi: 10.1016/0002-9610(80)90369-4, PMID: 7468923

[6] Lee IS Ahn JY Yook JH Kim BS . Mediastinal lymph node dissection and distal esophagectomy is not essential in early esophagogastric junction adenocarcinoma. World J Surg Oncol. (2017) 15:28–35. doi: 10.1186/s12957-016-1088-x, PMID: 28100248 PMC5242091

[7] Rüdiger Siewert J Feith M Werner M Stein HJ . Adenocarcinoma of the esophagogastric junction: results of surgical therapy based on anatomical/topographic classification in 1,002 consecutive patients. Ann Surg. (2000) 232:353–61. doi: 10.1097/00000658-200009000-00007, PMID: 10973385 PMC1421149

[8] Parry K Haverkamp L Bruijnen RC Siersema PD Ruurda JP van Hillegersberg R . Surgical treatment of adenocarcinomas of the gastro-esophageal junction. Ann Surg Oncol. (2015) 22:597–603. doi: 10.1245/s10434-014-4047-1, PMID: 25190126

[9] Voron T Gronnier C Pasquer A Thereaux J Gagniere J Lebreton G . Adenocarcinoma of the oesophagogastric junction Siewert II: An oesophageal cancer better cured with total gastrectomy. Eur J Surg Oncol. (2019) 45:2473–81. doi: 10.1016/j.ejso.2019.07.022, PMID: 31350076

[10] Mazer LM Poultsides GA . What is the best operation for proximal gastric cancer and distal esophageal cancer? Surg Clin North Am. (2019) 99:457–69. doi: 10.1016/j.suc.2019.02.003, PMID: 31047035

[11] Hulscher JB van Sandick JW de Boer AG Wijnhoven BP Tijssen JG Fockens P . Extended transthoracic resection compared with limited transhiatal resection for adenocarcinoma of the esophagus. N Engl J Med. (2002) 347:1662–9. doi: 10.1056/NEJMoa022343, PMID: 12444180

[12] Sasako M Sano T Yamamoto S Sairenji M Arai K Kinoshita T . Left thoracoabdominal approach versus abdominal-transhiatal approach for gastric cancer of the cardia or subcardia: a randomized controlled trial. Lancet Oncol. (2006) 7:644–51. doi: 10.1016/S1470-2045(06)70766-5, PMID: 16887481

[13] Wei MT Zhang YC Deng XB Yang TH He YZ Wang ZQ . Transthoracic vs transhiatal surgery for cancer of the esophagogastric junction: a meta-analysis. World J Gastroenterol. (2014) 20:10183–92. doi: 10.3748/wjg.v20.i29.10183, PMID: 25110447 PMC4123349

[14] Kurokawa Y Hiki N Yoshikawa T Kishi K Ito Y Ohi M . Mediastinal lymph node metastasis and recurrence in adenocarcinoma of the esophagogastric junction. Surgery. (2015) 157:551–5. doi: 10.1016/j.surg.2014.08.099, PMID: 25532434

[15] Kinoshita T Gotohda N Kato Y Takahashi S Konishi M Okazumi S . Laparoscopic transhiatal resection for Siewert type II adenocarcinoma of the esophagogastric junction: operative technique and initial results. Surg Laparosc Endosc Percutan Tech. (2012) 22:e199–203. doi: 10.1097/SLE.0b013e31825a72e2, PMID: 22874699

[16] Sugita S Kinoshita T Kaito A Watanabe M Sunagawa H . Short-term outcomes after laparoscopic versus open transhiatal resection of Siewert type II adenocarcinoma of the esophagogastric junction. Surg Endosc. (2018) 32:383–90. doi: 10.1007/s00464-017-5687-6, PMID: 28656339

[17] Hosoda K Yamashita K Katada N Moriya H Mieno H Sakuramoto S . Benefit of neoadjuvant chemotherapy for Siewert type II esophagogastric junction adenocarcinoma. Anticancer Res. (2015) 35:419–25. 25550582

[18] Coccolini F Nardi M Montori G Ceresoli M Celotti A Cascinu S . Neoadjuvant chemotherapy in advanced gastric and esophago-gastric cancer. Meta-analysis of randomized trials. Int J Surg. (2018) 51:120–7. doi: 10.1016/j.ijsu.2018.01.008, PMID: 29413875

[19] Feng F Tian Y Xu G Liu S Liu Z Zheng G . The length of proximal margin does not influence the prognosis of Siewert type II/III adenocarcinoma of esophagogastric junction after transhiatal curative gastrectomy. Springerplus. (2016) 5:588. doi: 10.1186/s40064-016-2240-3, PMID: 27247885 PMC4864759

[20] Barbour AP Rizk NP Gonen M Tang L Bains MS Rusch VW . Adenocarcinoma of the gastroesophageal junction influence of esophageal resection margin and operative approach on outcome. Ann Surg. (2007) 246:1–8. doi: 10.1097/01.sla.0000255563.65157.d2, PMID: 17592282 PMC1899203

[21] Gao F Chen J Wang T Wang G Zhang Z Shen L . Incidence of microscopically positive proximal margins in adenocarcinoma of the gastroesophageal junction. PloS One. (2014) 9:e88010. doi: 10.1371/journal.pone.0088010, PMID: 24505353 PMC3914864

[22] Mine S Sano T Hiki N Yamada K Kosuga T Nunobe S . Proximal margin length with transhiatal gastrectomy for Siewert type II and III adenocarcinomas of the oesophagogastric junction. Br J Surg. (2013) 100:1050–4. doi: 10.1002/bjs.9170, PMID: 23754647

[23] Postlewait LM Squires MH 3rd Kooby DA Poultsides GA Weber SM Bloomston M . The importance of the proximal resection margin distance for proximal gastric cancer adenocarcinoma: A multi-institutional study of the US Gastric Cancer Collaborative. J Surg Oncol. (2015) 112:203–7. doi: 10.1002/jso.23971, PMID: 26272801

[24] Kim MG Lee JH Ha TK Kwon SJ . The distance of proximal resection margin dose not significantly influence on the prognosis of gastric cancer patients after curative resection. Ann Surg Treat Res. (2014) 87:223–31. doi: 10.4174/astr.2014.87.5.223, PMID: 25368847 PMC4217255

[25] Lee CM Jee YS Lee JH Son SY Ahn SH Park DJ . Length of negative resection margin does not affect local recurrence and survival in the patients with gastric cancer. World J Gastroenterol. (2014) 20:10518–24. doi: 10.3748/wjg.v20.i30.10518, PMID: 25132770 PMC4130861

[26] Niclauss N Jung MK Chevallay M Mönig SP . Minimal length of proximal resection margin in adenocarcinoma of the esophagogastric junction: a systematic review of the literature. Updates Surg. (2019) 71:401–9. doi: 10.1007/s13304-019-00665-w, PMID: 31243725

[27] Pang T Nie M Yin K . The correlation between the margin of resection and prognosis in esophagogastric junction adenocarcinoma. World J Surg Oncol. (2023) 21:316. doi: 10.1186/s12957-023-03202-7, PMID: 37814242 PMC10561513

[28] von Rahden BH Stein HJ Feith M Becker K Siewert JR . Lymphatic vessel invasion as a prognostic factor in patients with primary resected adenocarcinomas of the esophagogastric junction. J Clin Oncol. (2005) 23:874–9. doi: 10.1200/JCO.2005.12.151, PMID: 15681533

[29] Shen JG Cheong JH Hyung WJ Kim J Choi SH Noh SH . Influence of a microscopic positive proximal margin in the treatment of gastric adenocarcinoma of the cardia. World J Gastroenterol. (2006) 12:3883–6. doi: 10.3748/wjg.v12.i24.3883, PMID: 16804975 PMC4087938

[30] Gall CA Rieger NA Wattchow DA . Positive proximal resection margins after resection for carcinoma of the oesophagus and stomach: effect on survival and symptom recurrence. Aust N Z J Surg. (1996) 66:734–7. doi: 10.1111/j.1445-2197.1996.tb00732.x, PMID: 8918379

[31] St-Amour P Winiker M Sempoux C Fasquelle F Demartines N Schäfer M . The “Real R0”: A resection margin smaller than 0.1 cm is associated with a poor prognosis after oncologic esophagectomy. Ann Surg Oncol. (2021) 28:7095–106. doi: 10.1245/s10434-021-10121-y, PMID: 34041624 PMC8519834

[32] Juez LD Barranquero AG Priego P Cuadrado M Blázquez L Sánchez-Picot S . Influence of positive margins on tumour recurrence and overall survival after gastrectomy for gastric cancer. ANZ J Surg. (2021) 91:E465–73. doi: 10.1111/ans.16937, PMID: 34013576

[33] Yuan P Yan Y Jia Y Wang J Li Z Wu Q . Intraoperative gastroscopy to determine proximal resection margin during totally laparoscopic gastrectomy for patients with upper third gastric cancer. J Gastrointest Oncol. (2021) 12:142–52. doi: 10.21037/jgo-20-277, PMID: 33708432 PMC7944160

[34] Su PF Yu JC . Progress in neoadjuvant therapy for gastric cancer. Oncol Lett. (2022) 23:172. doi: 10.3892/ol.2022.13292, PMID: 35497934 PMC9019865

[35] Hartgrink HH van de Velde CJ Putter H Songun I Tesselaar ME Kranenbarg EK . Neo-adjuvant chemotherapy for operable gastric cancer: Long term results of the Dutch randomised FAMTX trial. Eur J Surg Oncol. (2004) 30:643–9. doi: 10.1016/j.ejso.2004.04.013, PMID: 15256239

[36] Schuhmacher C Gretschel S Lordick F Reichardt P Hohenberger W Eisenberger CF . Neoadjuvant chemotherapy compared with surgery alone for locally advanced cancer of the stomach and cardia: European Organisation for Research and Treatment of Cancer randomized trial 40954. J Clin Oncol. (2010) 28:5210–8. doi: 10.1200/JCO.2009.26.6114, PMID: 21060024 PMC3020693

[37] Clemente-Gutiérrez U Sánchez-Morales G Santes O Medina-Franco H . Clinical usefulness of extending the proximal margin in total gastrectomies for gastric adenocarcinoma. Rev Gastroenterol Mex (Engl Ed). (2019) 84:136–42. doi: 10.1016/j.rgmx.2018.03.006, PMID: 29753577

[38] Watanabe A Adamson H Lim H McFadden AF McConnell YJ Hamilton TD . Intraoperative frozen section analysis of margin status as a quality indicator in gastric cancer surgery. J Surg Oncol. (2023) 127:66–72. doi: 10.1002/jso.27107, PMID: 36177786

[39] Squires MH 3rd Kooby DA Pawlik TM Weber SM Poultsides G Schmidt C . Utility of the proximal margin frozen section for resection of gastric adenocarcinoma: a 7-Institution Study of the US Gastric Cancer Collaborative. Ann Surg Oncol. (2014) 21:4202–10. doi: 10.1245/s10434-014-3834-z, PMID: 25047464

[40] Gundavda K Rajavelu N Chopde A Rane P Ostwal V Ramaswamy A . Intraoperative revision of a positive proximal resection margin is associated with improved survival in patients undergoing resection for gastroesophageal junction and proximal third gastric adenocarcinoma. J Gastrointest Surg. (2025) 29:102123. doi: 10.1016/j.gassur.2025.102123, PMID: 40545154

